# Enhancing Mucoadhesive Properties of Gelatin through Chemical Modification with Unsaturated Anhydrides

**DOI:** 10.1021/acs.biomac.3c01183

**Published:** 2024-02-06

**Authors:** Elvira O. Shatabayeva, Daulet B. Kaldybekov, Leila Ulmanova, Balnur A. Zhaisanbayeva, Ellina A. Mun, Zarina A. Kenessova, Sarkyt E. Kudaibergenov, Vitaliy V. Khutoryanskiy

**Affiliations:** † Reading School of Pharmacy, University of Reading, Whiteknights, RG6 6DX Reading, United Kingdom; ‡ Department of Chemistry and Chemical Technology, Al-Farabi Kazakh National University, 050040 Almaty, Kazakhstan; § Institute of Polymer Materials and Technology, 050019 Almaty, Kazakhstan; ∥ School of Sciences and Humanities, Nazarbayev University, 010000 Astana, Kazakhstan; ⊥ School of Engineering and Digital Sciences, Nazarbayev University, 010000 Astana, Kazakhstan

## Abstract

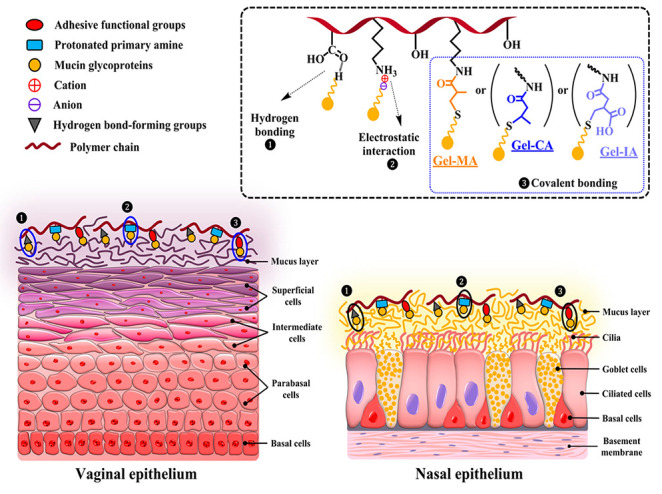

Gelatin is a water-soluble natural polyampholyte with poor mucoadhesive properties. It has traditionally been used as a major ingredient in many pharmaceuticals, including soft and hard capsules, suppositories, tissue engineering, and regenerative medicine. The mucoadhesive properties of gelatin can be improved by modifying it through conjugation with specific adhesive unsaturated groups. In this study, gelatin was modified by reacting with crotonic, itaconic, and methacrylic anhydrides in varying molar ratios to yield crotonoylated-, itaconoylated-, and methacryloylated gelatins (abbreviated as Gel-CA, Gel-IA, and Gel-MA, respectively). The successful synthesis was confirmed using ^1^H NMR, FTIR spectroscopies, and colorimetric TNBSA assay. The effect of chemical modification on the isoelectric point was studied through viscosity and electrophoretic mobility measurements. The evolution of the storage (*G*′) and loss (*G*′′) moduli was employed to determine thermoreversible gelation points of modified and unmodified gelatins. The safety of modified gelatin derivatives was assessed with an *in vivo* slug mucosal irritation test (SMIT) and an *in vitro* MTT assay utilizing human pulmonary fibroblasts cell line. Two different model dosage forms, such as physical gels and spray-dried microparticles, were prepared and their mucoadhesive properties were evaluated using a flow-through technique with fluorescent detection and a tensile test with *ex vivo* porcine vaginal tissues and sheep nasal mucosa. Gelatins modified with unsaturated groups exhibited superior mucoadhesive properties compared to native gelatin. The enhanced ability of gelatin modified with these unsaturated functional groups is due to the formation of covalent bonds with cysteine-rich subdomains present in the mucin via thiol–ene click Michael-type addition reactions occurring under physiologically relevant conditions.

## 1 Introduction

Gelatin is a natural biopolymer derived from collagen through its partial hydrolysis and heat denaturation. Collagen itself is extracted from the bones, cartilage, connective tissues, and skins of slaughtered animals, and fish scales.^1^ It is industrially one of the most important and widely used polyampholytes owing to its unique physicochemical properties. The properties of gelatin depend on the method of its production, which can involve either acid or alkaline treatment. Commercially available type A gelatin, produced via the acid process, has an isoelectric point (IEP) at pH 7–9, whereas type B gelatin, manufactured through the alkaline process, exhibits an IEP at pH 4.5–5.5. As a protein-based biomaterial, gelatin has excellent biocompatibility, biodegradability, nontoxicity, and nonimmunogenicity. It has been approved as a GRAS (generally recognized as safe) material by the U.S. Food and Drug Administration (FDA). Gelatin readily dissolves in warm water at ≥35 °C and has the ability to form physically cross-linked thermoreversible hydrogels upon cooling below ∼23 °C. Additionally, it exhibits a melting point close to body temperature. These distinctive properties are the key factors driving its extensive applications in pharmaceutical, food, and cosmetic industries.^1−3^ Gelatin is commonly used as a major ingredient in many formulations, including hard and soft capsules,^4^ vaginal and rectal suppositories,^5^ as matrices in tissue engineering and regenerative medicine,^6^ as a carrier in drug delivery (e.g., microspheres),^7^ and in many other health-related applications.^8,9^

Mucoadhesion is defined as the ability of materials to adhere to mucosal membranes in the body for an extended period of time. The established routes for transmucosal drug administration include ocular (corneal and conjunctival), nasal, oromucosal (buccal and sublingual), gastrointestinal, rectal, vaginal, and intravesical. Transmucosal drug delivery offers several important advantages, such as the ease of dosage form administration and its noninvasive nature, prolonged residence time on the mucosal surface, improved drug bioavailability, the avoidance of hepatic first-pass metabolism, reduced dosing frequency, and the possibility for quick termination of therapy when needed.^10^

Numerous first-generation (conventional) mucoadhesive polymers are traditionally used in various dosage forms for transmucosal drug delivery. This class of mucoadhesives includes water-soluble polymers of both natural and synthetic origins, such as chitosan, gellan gum, alginate, polycarbopols, and cellulose derivatives (e.g., carboxymethylcellulose, hydroxypropyl methylcellulose, and others). The mucoadhesion in these drug delivery systems occurs through various physical interactions (nonspecific binding) between the macromolecules and mucin glycoproteins present on mucosal surfaces. These interactions primarily involve noncovalent forces, such as hydrogen bonding, electrostatic attraction, van der Waals forces, hydrophobic interactions, and chain entanglements/diffusion. Generally, polyelectrolytes exhibit better mucoadhesive properties compared to nonionic polymers. Furthermore, the mechanisms of adhesion of these materials to mucosal surfaces may vary depending on the nature of the dosage form. For instance, adhesive properties of solid dosage forms (such as tablets) are influenced by the hydration process, while the retention of liquid formulations on mucosal surfaces is more affected by their rheological properties.

In the past few decades, different chemical approaches have been explored to design polymers and their formulations with enhanced mucoadhesive properties.^11^ This enhancement can be accomplished through functionalization of hydrophilic polymers with specific adhesive groups capable of forming covalent bonds with mucosal tissues under physiological conditions. Thiolated polymers, also known as “thiomers”, represent one of the prominent advances in the second-generation mucoadhesive materials, pioneered by Bernkop-Schnürch and co-workers.^12^ These polymers have been modified by introducing thiol (sulfhydryl) functional groups onto their side chains. Thiolated polymers form interdisulfide bridges through covalent interactions (via oxidation reactions) with cysteine-bearing subdomains of mucus glycoproteins present on mucosal surfaces. This leads to their enhanced mucoadhesive capabilities resulting in a prolonged drug residence time at the side of application.^13,14^ However, it is worth noting that thiolated polymers are prone to oxidation, which can lead to unwanted cross-linking of polymers.

Various strategies have recently been proposed to enhance the mucoadhesive properties of hydrophilic polymers by introducing adhesive moieties such as acryloyl,^15^ methacryloyl,^16^ maleimide,^17^ catechol,^18^ boronate,^19^ and *N*-hydroxy(sulfo)succinimide ester groups.^20^ Acryloylated polymers, first proposed by the Bianco-Peled research group,^15,21^ were highlighted as a novel class of pharmaceutical excipients with substantially improved mucoadhesive properties compared to their unmodified counterparts. The Khutoryanskiy group also pioneered the use of methacryloyl- and maleimide-functionalized materials to design dosage forms with enhanced mucoadhesive properties. These include the development of nanogels,^17^ liposomes,^22^ nanoparticles,^23^ liquid formulations,^24^*in situ* gels,^25^ and spray-coated tablets.^26^ These unsaturated functional groups are able to form covalent bonds with cysteine-rich subdomains of mucin glycoproteins through thiol–ene click Michael-type addition reactions to achieve strong mucoadhesive bonds. Moreover, (meth)acryloyl- and maleimide-functionalized macromolecules have potentially better stability against oxidation with no tendency for inter- and intramolecular cross-linking unlike thiolated polymers. Recently, aldehyde-functionalized nanocarriers have also been shown the ability to adhere to the porcine urinary bladder and sheep nasal mucosae strongly by forming imine bonds with primary amine groups present on the surface of mucosal tissue via Schiff base chemistry.^27,28^

Traditionally, gelatin has been regarded as a polymer with poor mucoadhesive properties. Gelatin’s adhesion capabilities are primarily attributed to its amphoteric nature, which results in weak electrostatic interactions with mucosal surfaces when compared to strong covalent interactions. The findings regarding the bio/mucoadhesive properties of gelatin have been a subject of debate. Several studies have reported that gelatin exhibits favorable mucoadhesiveness, or addition of gelatin improves the adhesive properties of the studied systems to some extent.^29−32^ However, most studies report that pristine gelatin demonstrates weak mucoadhesive properties, and/or its presence does not significantly contribute to the adhesiveness of formulations.^33−38^ Nevertheless, gelatin contains reactive sites within its molecular structure, such as amine, carboxylic, and hydroxyl groups, providing opportunities for conjugation with functional groups that can lead to changes in its physical and chemical properties. A recent review by Ahmady and Abu Samah^39^ discussed various strategies to enhance the mucoadhesive properties of gelatin. One suggested approach is the introduction of methacryloyl moieties into gelatin; however, as far as we are aware, there is no conclusive evidence supporting its positive impact on mucoadhesive properties.

In the present study, we report the modification of gelatin by the reactions with three unsaturated anhydrides (crotonic, itaconic, and methacrylic anhydrides) in order to enhance its mucoadhesive properties. The resulting gelatin derivatives were fully characterized using ^1^H NMR and FTIR spectroscopies and colorimetric TNBSA assay. The effect of chemical modification on the isoelectric point was studied using viscosity and electrophoretic mobility measurements. The evolution of the storage (*G*′) and loss (*G*′′) moduli was employed to determine the thermo-reversible gelation points of both modified and unmodified gelatins. The toxicological properties of these derivatives were also assessed using *in vivo* slug mucosal irritation test (SMIT) and *in vitro* MTT assay in human pulmonary fibroblasts cell line. Two different model dosage forms such as physical gels and spray-dried microparticles were prepared and their retention on mucosal surfaces were evaluated using *ex vivo* porcine vaginal and sheep nasal mucosae.

## 2 Experimental Section

### 2.1 Materials

Crotonic anhydride (CA), itaconic anhydride (IA), methacrylic anhydride (MA), type A gelatin from porcine skin (gel strength ∼175 g Bloom), benzalkonium chloride (BAC), deuterium oxide, 3-(4,5-dimethylthiazol-2-yl)-2,5-diphenyltetrazolium bromide (MTT reagent), fluorescein sodium salt (NaFl), glutaraldehyde solution (25% in H_2_O, grade II), glycine, 1 M hydrochloric acid solution, sodium bicarbonate, sodium dodecyl sulfate (SDS), 2,4,6-trinitrobenzenesulfonic acid (TNBSA, 5% in H_2_O), and trypan blue (0.4% solution) were purchased from Sigma-Aldrich (Gillingham, U.K.). Dimethyl sulfoxide (DMSO), Dulbecco’s modified eagle medium (DMEM), fetal bovine serum (FBS), penicillin-streptomycin (10000 U/mL), phosphate-buffered saline (PBS) tablets (which were used to make 100 mL of 1× PBS solution in deionized water, pH 7.40), sodium hydroxide, and trypsin-EDTA (0.25% solution) were purchased from Fisher Scientific (Loughborough, U.K.). All other chemicals were of analytical grade and used without further purification. Dialysis tubing with a molecular weight cutoff of 12–14 kDa was purchased from Medicell Membranes Ltd. (London, U.K.). Deionized water was used throughout the experiments involving aqueous solutions.

### 2.2 Synthesis of Gelatin Derivatives

Gelatin was chemically functionalized with different unsaturated anhydrides using previously described procedures with some modifications.^40,41^ Briefly, gelatin (0.5 g) was dissolved in 100 mL of PBS solution (pH 7.40) at 50 °C until a transparent homogeneous solution formed while stirred. Subsequently, desired amounts of crotonic anhydride, itaconic anhydride, or methacrylic anhydride were added dropwise to the gelatin solutions and reacted for 12 h at 50 °C under constant stirring to produce crotonoylated, itaconoylated or methacryloylated gelatin derivatives (abbreviated as Gel-CA, Gel-IA, and Gel-MA, respectively). The pH was maintained at 8.50 throughout the reaction by adding 4 M NaOH solution. Following the dilution with an additional 50 mL of PBS solution (pH 7.40) to quench the reaction, each resulting product was then purified by dialysis against deionized water (5 L; water changed 8 times) for 48 h in the dark to remove salts, unreacted anhydrides, and byproduct acids. Finally, the solution was lyophilized, forming a white sponge-like product, sealed and stored in a freezer until further use. The data on the varied amounts of anhydrides present in the initial reaction mixture are summarized in Table S1 in the Supporting Information.

### 2.3 Preparation of Spray-Dried Microparticles

Both chemically modified and unmodified gelatin samples (0.5 g) were initially dissolved in 100 mL of aqueous solutions containing fluorescein sodium salt (1 mg/mL) at 40 °C for 60 min while stirring at 400 rpm. In separate preparation, 200 μL of 25% glutaraldehyde aqueous solutions were added into gelatin-based solutions and stirred for another 60 min at 400 rpm. Subsequently, the resulting solutions were spray-dried using a Büchi Mini Spray Dryer B-290 (Büchi Labortechnik AG, Flawil, Switzerland) equipped with a Dehumidifier S-396 to generate free-flowing cross-linked and non-cross-linked NaFl-loaded gelatin-based microparticles. The solutions were delivered to the nozzle at a feed rate of 5 mL/min using a peristaltic pump and then spray-dried at 140 °C inlet temperature and outlet temperature of 75 °C. As a standard, the aspirator rate was set to 100% to maximize the separation rate of the cyclone; compressed nitrogen was used to disperse the liquid into fine droplets. The resultant spray-dried products were collected, sealed to protect from rehydration, wrapped with aluminum foil, and stored in a freezer until further tests.

### 2.4 Characterization

#### 2.4.1 Quantification of the Degree of Functionalization

The modification of gelatin was confirmed using ^1^H NMR spectroscopy. Twenty mg/mL of gelatin and its derivatives (Gel-CA, Gel-IA, and Gel-MA) were dissolved in warm D_2_O. ^1^H NMR spectra of samples were recorded using a 500 MHz Bruker Avance III NMR spectrometer (Bruker UK Ltd., Coventry, U.K.) at 37 °C with 128 scans per spectrum. Prior to interpretation, each resulting spectrum was phase corrected. Baseline correction was applied before integrating the signals of interest.

^1^H NMR analysis was employed to quantify the degree of functionalization (DoF) of gelatin derivatives similar to that described previously.^42−44^ The percentage of unsaturated groups incorporation was estimated by comparing the integrated intensities of the double bond peaks to those of the aromatic residues present in gelatin side chains.

A colorimetric 2,4,6-trinitrobenzenesulfonic acid (TNBSA) assay, originally developed by Habeeb, was also used to determine the remaining free amino groups after gelatin derivatization with minor changes.^45−47^ Briefly, gelatin and its derivatives (1 mg/mL) were separately dissolved in 0.1 M sodium bicarbonate solution (pH 8.50). Then, 500 μL of 0.1% v/v TNBSA solution (prepared in 0.1 M NaHCO_3_ buffer) was added to 500 μL of each test sample solution and incubated at 37 °C for 2 h with gentle stirring. Afterward, 500 μL of 10% w/v sodium dodecyl sulfate (SDS) and 250 μL of 1 M HCl were added to each test sample to stop the reaction. The absorbance of each solution was then measured at 335 nm and the concentration of free primary amines was quantified using a glycine standard curve (see Figure S1 in the Supporting Information). The amount of free amino groups was determined to be 0.434 mmol per 1 g of gelatin. The DoF was calculated by subtracting the amount of remaining free −NH_2_ groups in each modified gelatin from the amount of free −NH_2_ groups in native gelatin. A UV-1900i Shimadzu UV–vis spectrophotometer (Kyoto, Japan) was employed to record the UV–vis absorption spectra of the samples. All experiments with DoF quantification were conducted in triplicate.

#### 2.4.2 Fourier Transform Infrared (FTIR) Spectroscopy

FTIR spectra of unmodified and modified gelatins were recorded using a Nicolet iS10 FTIR spectrophotometer (Thermo Scientific, U.K.) with an iTX attenuated total reflectance (ATR) accessory equipped with a diamond crystal. The spectra were collected from an average of 32 scans between 4000 and 500 cm^–1^, employing the transmittance mode with a spectral resolution of 4 cm^–1^.

#### 2.4.3 Scanning Electron Microscopy (SEM)

The morphology and size of spray-dried microparticles based on gelatin and its modified (Gel-CA, Gel-IA, and Gel-MA) derivatives were examined using a Zeiss Crossbeam 540 scanning electron microscope (Carl Zeiss Microscopy GmbH, Jena, Germany) at an accelerating voltage of 5 kV. The samples were sputter-coated with gold prior to imaging. The acquired images were then analyzed with ImageJ software (NIH, U.S.A.) to determine the average mean diameter of the microparticles.

#### 2.4.4 Determination of the Isoelectric Point (IEP)

The isoelectric points of modified and unmodified gelatin were determined using a conventional viscometric technique^48^ and electrophoretic mobility using a Zetasizer Nano-ZS90 instrument (Malvern Instruments, Worcestershire, U.K.), respectively, while varying the solution pH. All measurements were carried out in triplicates at 25 °C. Briefly, a 1% (w/v) solution of either gelatin or its derivatives was prepared in deionized water at 40 °C and stirred until complete dissolution. The specific viscosity was determined using an Ostwald-type capillary viscometer (with capillary diameter of 0.86 mm) and is expressed as the ratio of the time of flow (*t*) to that of water (*t*_0_):
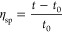
1The isoelectric point (IEP_viscometry_) of each sample was determined based on the pH at which the polymer solution exhibited a minimum viscosity value, indicating that the overall charge of the macromolecules is close to zero at that specific pH.

In electrophoretic mobility experiments, a typical protein refractive index of 1.45 and absorbance of 0.001 were used for all measurements in zeta-potential mode. Viscosity (0.8872 cP) and refractive index (1.33) of water were used as dispersant parameters. Each sample was analyzed three times. The results were processed using the Smoluchowski model (Fκa = 1.50), and the average electrophoretic mobility mean ± standard deviation values were calculated. The electrophoretic mobility versus pH curve was plotted for each test sample and the point at which the curve intersects zero mobility was considered as an IEP_EM_.

#### 2.4.5 Rheological Studies

A TA DHR-1 rheometer (TA Instruments, New Castle, DE, U.S.A.) equipped with a variable temperature Peltier plate and a stainless steel cone–plate geometry (⌀ = 40 mm; cone angle = 2°) was used to conduct the rheological experiments. The experiments aimed to determine the gelation and melting temperatures of gelatin samples. Initially, the samples (5% w/v aqueous solutions) were equilibrated at 40 °C for 5 min. Subsequently, each sample was cooled from 40 to 0 °C, followed by temperature sweep tests from 0 to 40 °C (heated) and then reduced back to 0 °C (cooled) at a scanning rate of 2 °C/min. During these tests, the changes in the storage modulus (*G*′) and loss modulus (*G*′′) were recorded as a function of temperature at an applied strain of 1% and a frequency of 10 rad/s (equivalent to 1.6 Hz). A solvent trap cover was used to ensure uniform temperature and prevent evaporation of the mixture. The results obtained were calculated as the mean values of 3 measurements.

### 2.5 Toxicity Assessment

#### 2.5.1 *In Vitro* Cell Viability Assay

Human pulmonary fibroblasts (HPF) cell line was cultured in Dulbecco’s modified eagle medium (DMEM) fortified with 10% fetal bovine serum (FBS) and 1% streptomycin/penicillin at 37 °C in a humidified incubator with 5% CO_2_. Upon reaching 75% confluency in T-25 cell culture flasks, cells were harvested using 0.25% trypsin-EDTA solution, seeded in 96-well plates at a density of 5 × 10^3^ cells per well, and kept overnight under standard cultivation conditions (37 °C and 5% CO_2_ in the humidified incubator) to promote cell attachment. The cells were then treated with modified and unmodified gelatin solutions (Gel-CA, Gel-IA, and Gel-MA,) at concentrations of 1.3 and 5% (w/v) in cell growth medium. The cells were then incubated for 24 h at 37 °C in a humidified 5% CO_2_ incubator. Nontreated cells and 10% DMSO solution were used as a negative and a positive control, respectively. Positive control was used to check the assay activity. Following the exposure to the solutions of gelatin and its derivatives, each well received 10 μL of MTT solution (5 mg/mL) in the dark, and the cells were further incubated for 4 h at 37 °C in a humidified 5% CO_2_ incubator. Formazan crystals produced during the incubation period were dissolved by adding 10% (w/v) sodium dodecyl sulfate (SDS). The absorbance was measured at 570 nm with a Varioskan microplate reader (Thermo Scientific, U.S.A.). The viability percentage was calculated using the following equation:
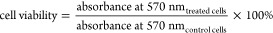
2

#### 2.5.2 Slug Mucosal Irritation Test

Slug mucosal irritation test (SMIT) was conducted *in vivo* using the methodology previously described by our research group.^49^*Arion lusitanicus* slugs were collected in Harris Garden (Reading, U.K.) and housed in ventilated plastic containers at room temperature. They were fed with lettuce, cabbage, and cucumber for 48 h. Then, the body lining of each slug was carefully inspected, and only slugs without macroscopic injuries with clear tubercles and a foot surface were used for testing. Slugs weighing ∼5–16 g were then selected and kept individually in 2 L glass beakers lined with a paper towel moistened with 20 mL of PBS solution (pH 7.40) and left at room temperature for 48 h before commencing the experiments. The beakers were covered with punctured cling film to ensure air exchange. Then, each slug was individually weighed and placed in a 90 mm plastic Petri dish lined with Whatman filter paper soaked with 2 mL of sample solutions. The test samples included PBS solution (negative control), 1% w/v benzalkonium chloride (positive control) prepared in PBS, as well as 1.3% (w/v) of modified and unmodified gelatin solutions prepared in PBS. Following 60 min contact with test samples, slugs were removed from the Petri dishes, rinsed with 10 mL of PBS, gently wiped with the paper towel, and then reweighed. The mucus production (MP) was estimated based on a slug body weight loss and calculated using the following equation:
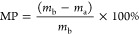
3where *m*_b_ and *m*_a_ are the weights of a slug before and after exposure to the tested materials, respectively. The results of the experiments were expressed as mean ± standard deviation values (*n* = 7) and evaluated statistically.

### 2.6 *Ex Vivo* Retention Studies on Porcine Vaginal and Sheep Nasal Mucosae

#### 2.6.1 Model Dosage Form Design

A 0.1 mg/mL NaFl solution was prepared in deionized water and used as a model drug compound. Then, 0.5 g (5% w/v) of gelatin and its crotonoylated (Gel-CA), itaconoylated (Gel-IA), and methacryloylated (Gel-MA) derivatives were separately dissolved in 10 mL of aqueous solutions of NaFl. The mixtures were stirred for 12 h at room temperature until homogeneous solutions formed, covered with aluminum foil, and stored in a fridge for further use. These NaFl-loaded modified and unmodified gelatin-based formulations were employed in porcine vaginal mucoadhesion studies.

The preparation of NaFl-loaded spray-dried microparticles based on gelatin and its derivatives is described in section 2.3. These microparticles were used in sheep nasal mucoadhesion studies. The formulations to prepare vaginal fluid simulant (VFS; pH 4.0) and artificial nasal fluid (ANF; pH 5.80) used for washing the mucosal surfaces are described in Tables S2 and S3 in the Supporting Information, respectively. VFS and ANF solutions were maintained at 37 °C throughout mucoadhesion experiments using a water bath.

#### 2.6.2 Tissue Preparation

Porcine vaginal tissues and sheep heads were received from P.C. Turner Abattoirs (Farnborough, U.K.) immediately after animal slaughter, packed, transported to the laboratory in cold plastic containers, and used within 24 h of collection. The vaginal tissues were carefully dissected (avoiding contact with the internal mucosa) using disposable sharp blades to yield ≈2 × 2 cm sections.

The nasal septum mucosal tissues were carefully extracted from sheep heads with bone-cutting shear scissors and then cut into a 1 × 1 cm square pieces with disposable sharp blades.

#### 2.6.3 Flow-through Technique

Experiments to evaluate the mucosal retention of modified and unmodified gelatin-based formulations on *ex vivo* porcine vaginal and sheep nasal tissues were conducted using a well-established flow-through method involving fluorescent detection with minor modifications.^22,50−53^ Initially, freshly excised vaginal or nasal tissue was mounted on a microscope glass slide with the mucosal side facing upward, then placed on a substrate fixed at an angle of 20° and prerinsed with 1 mL of either VFS or ANF solution, respectively, before commencing each *ex vivo* mucoadhesion test.

Fluorescence images were captured for the mucosal surface of the vaginal tissues using a Leica MZ10F stereomicroscope (Leica Microsystems, U.K.) equipped with a Leica DFC3000G digital camera fitted with a green fluorescence protein (GFP) filter (blue, λ_emission_ = 520 nm) at 1.25× magnification, with an exposure time of 10 ms and a 1.0× gain. Initially, images of blank vaginal tissues were acquired to determine the background fluorescence intensity for each sample prior to administration of the test material. Subsequently, prewarmed aliquots (200 μL) from either 5% w/v gelatin, Gel-CA, Gel-IA, or Gel-MA derivatives prepared in deionized water containing 0.1 mg/mL NaFl or a control of 0.1 mg/mL NaFl solution were deposited onto the mucosal surface. The samples were then repeatedly irrigated with VFS solution (pH 4.0) at a flow rate of 300 μL/min using a syringe pump. The fluorescence microscopy images of the mucosal surface of each vaginal sample were acquired at predetermined time points and then analyzed with ImageJ software (NIH, U.S.A.) by measuring the pixel intensity after each wash with VFS. The pixel intensity of the bare samples (vaginal mucosa without fluorescent test material) was subtracted from each measurement and data were converted into normalized intensity values using the following equation:
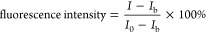
4where *I*_b_ is the background fluorescence intensity of a given tissue sample (a blank tissue); *I*_0_ denotes the initial fluorescence intensity of that sample (the tissue sample with a mucoadhesive fluorescent material applied on it before the start of first washing; this was considered as zero time point with 100% fluorescence intensity); and *I* represents the fluorescence intensity of that tissue sample with the mucoadhesive fluorescent material after each washing cycle. These fluorescence intensities were then converted into % mucosal retention values.

In addition, the mucoadhesive performance of spray-dried modified and unmodified gelatin-based microparticles was assessed using the same *in vitro* flow-through technique as described above with some modifications. Only non-cross-linked microparticle samples were used in this experiment. Approximately 100 mg of gelatin-based microparticles (included gelatin, Gel-CA, Gel-IA, or Gel-MA) containing 1 mg/mL NaFl were applied onto *ex vivo* sheep nasal mucosa, which was already mounted on a glass slide, placed on half-cut Falcon centrifuge tube inclined at an angle of 20°. ANF solution (pH 5.80) was then dripped onto the nasal mucosa at a flow rate of 200 μL/min using a syringe pump (total washing time was 30 min). The flow rate mentioned was intentionally set higher than the physiological production rate of nasal fluid for practical reasons. This adjustment was made to expedite the experiments and ensure they could be conducted within a reasonable time frame. Subsequently, ANF solution flowing through the nasal mucosa was collected at predetermined time intervals. Aliquots from a series of collected ANF solutions after each washing cycle were taken for analysis to determine the amount of NaFl-loaded microparticles that washed off the mucosal surface. The analysis was performed using a Varian Cary Eclipse fluorescence spectrophotometer (Santa Clara, CA, U.S.A.) at λ_excitation_ = 460 nm and λ_emission_ = 512 nm. The quantification was based on a NaFl standard curve (see Figure S2 in the Supporting Information) through the calculations, which provided the amount of retained formulations on sheep nasal mucosa.

#### 2.6.4 Tensile (Detachment) Method

A TA.XT Plus Texture Analyzer (Stable Micro Systems Ltd., Surrey, U.K.) operated in its adhesive test mode was used to evaluate the mucoadhesive performance of spray-dried microparticles based on gelatin and its derivatives. Both cross-linked and non-cross-linked microparticle samples were employed in this experiment to evaluate the contribution of macromolecules diffusion and ability to form interpenetrating layer with mucus in mucoadhesion. Freshly isolated sheep nasal tissues were used within 24 h of retrieval for this experiment. As previously reported and adapted with some changes,^10,54^ a section of sheep nasal tissue with the mucosal side facing downward was secured at the surface of a bespoke cylindrical probe. This probe was subsequently attached to the mobile arm of the texture analyzer. Another piece of nasal tissue with the mucosal side facing upward was securely mounted on the mucoadhesion rig of the texture analyzer. Prior to each measurement, nasal tissues were prerinsed with 1 mL of ANF solution (pH 5.80). Subsequently, ∼100 mg of each spray-dried gelatin-based microparticle formulation was dosed to the nasal mucosa mounted on the rig. The mobile cylindrical probe bearing the blank nasal tissue was then lowered to establish a contact with the opposing mucosal surface. Data acquired from the detachment experiments were used to assess the mucoadhesive strength and the total work of adhesion. The following equipment settings were applied: prespeed test 0.5 mm/sec; test speed 0.5 mm/sec; post-test speed 10 mm/sec; applied force 100 g (1 N); contact time 120 s; trigger type was “auto”; trigger force 5.0 g (0.049 N); and return distance 10 mm.

All the experiments to assess the retention of formulations on *ex vivo* porcine vaginal mucosa and sheep nasal mucosa were conducted at 37 °C and 100% relative humidity within an incubator to mimic physiological conditions. The measurements were all performed in triplicate, and the mean ± standard deviation values were calculated and then evaluated statistically.

### 2.7 Statistical Analysis

All measurements in the present study were conducted at a minimum of three times and data were expressed as mean ± standard deviation values. Statistical analyses were performed using a GraphPad Prism software (version 8.0; San Diego, CA, U.S.A.). Data were compared and assessed for significance using two-tailed Student’s *t*-test and a one-way analysis of variance (ANOVA) with Bonferroni *post hoc* test (for results of SMIT assay and mucoadhesion experiments). For the MTT assay, a one-way ANOVA with Dunnett’s multiple comparisons was employed to compare control and treated groups, while within the treated groups, two-tailed Student’s *t*-test was applied to compare gelatin and its derivatives. Statistical differences were considered significant at a level of *p* ≤ 0.05.

## 3 Results and Discussion

### 3.1 Synthesis and Characterization

**Scheme 1:**
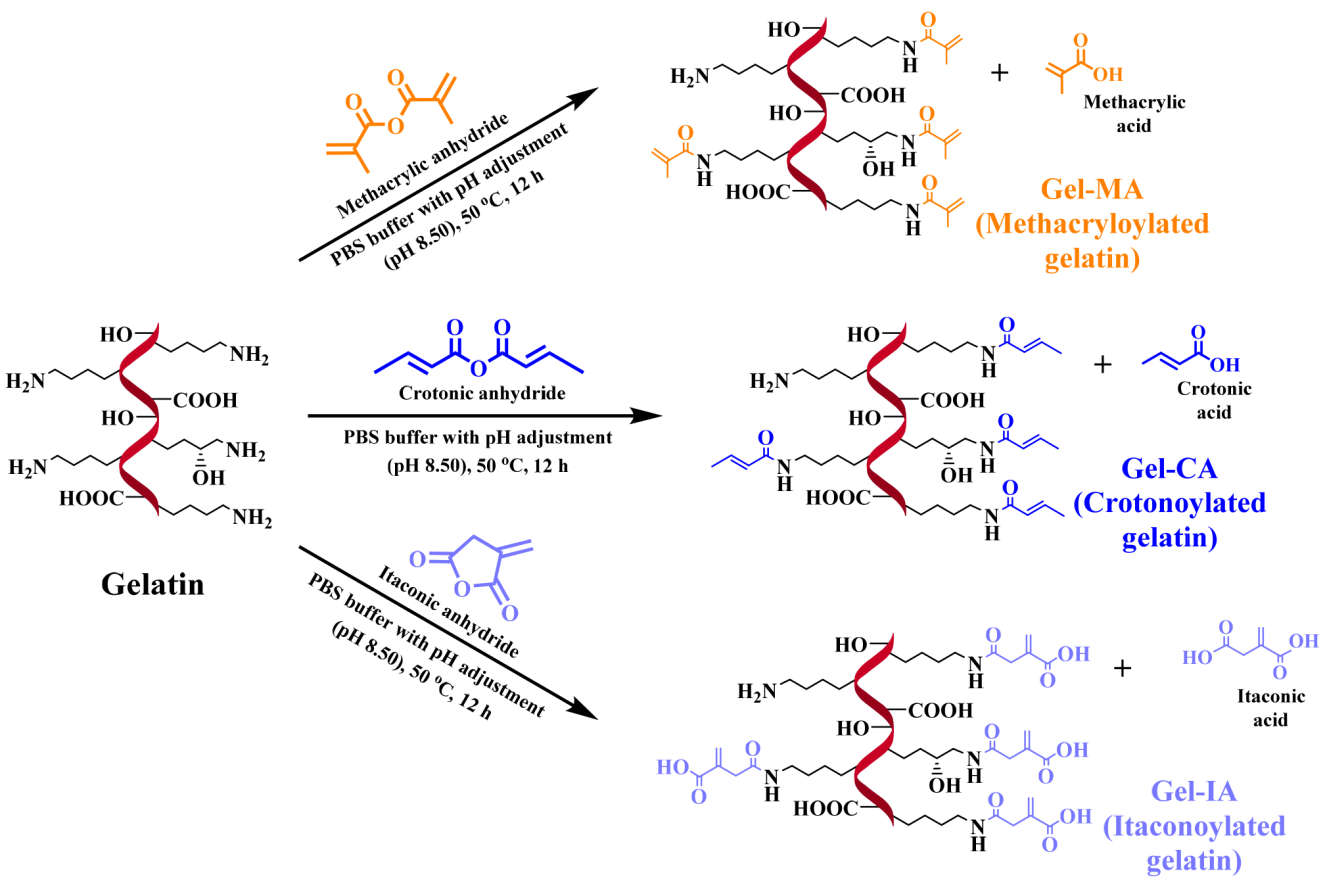
Schematic Illustration of the Modification Reaction of Gelatin with Different Unsaturated Anhydrides Please note that the schematic structure displays only two possibilities of an anhydride reaction with primary amine (−NH_2_) groups of lysine/hydroxylysine (which are the reactive sides) in the gelatin backbone. In reality, it could also react with any hydroxyl (−OH) groups present in gelatin due to increased reaction time and could result in a greater DoF.

**Figure 1:**
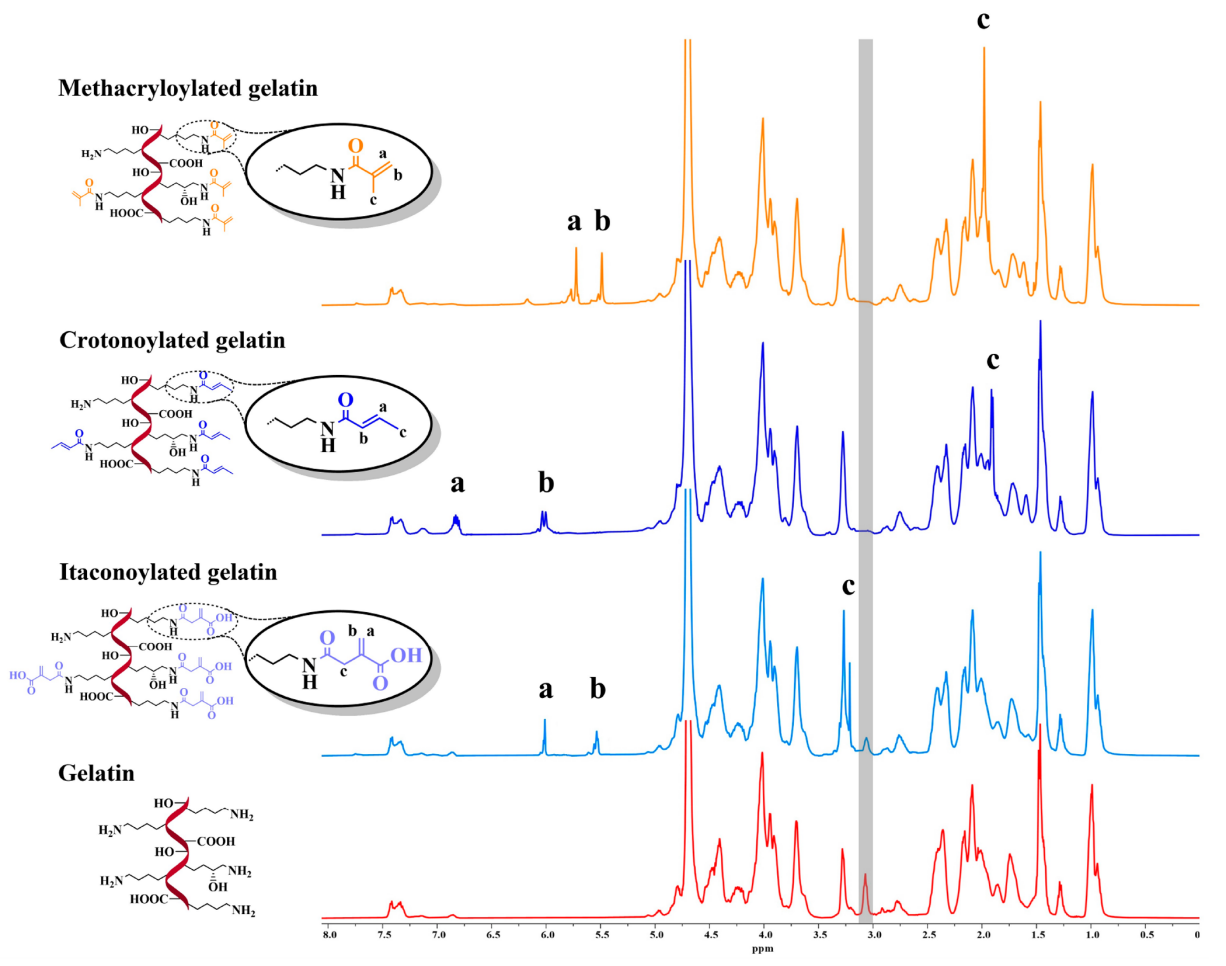
^1^H NMR spectra of modified and unmodified gelatins recorded in D_2_O at 37 °C.

The presence of reactive sites in gelatin macromolecules, such as amines and carboxylic and hydroxyl groups, provide an opportunity for chemical modification leading to changes in its physical and chemical properties. In this work, crotonoylated, itaconoylated, and methacryloylated gelatins were synthesized by reacting this biopolymer with crotonic, itaconic, and methacrylic anhydrides, respectively, at various feed ratios with respect to free amines on the gelatin backbone (Scheme 1). Following purification by dialysis and lyophilization, gelatin derivatives were then characterized using ^1^H NMR spectroscopy (Figure 1). All four spectra displayed the characteristic signals corresponding to the protons of aromatic amino acids in gelatin at ∼7.00 to 7.50 ppm. The degree of functionalization (DoF) was determined by comparing the integrals of the characteristic double bond hydrogen peaks of each modified gelatin substituent and the integration of the area corresponding to the combined peaks of the aromatic protons of phenylalanine and tyrosine, where their signals were served as a reference. Based on ^1^H NMR analysis of the spectra of modified gelatins new signals can be observed: protons from distinctive methylidene (**CH**_**2**_=C(CH_3_)-CONH−) group appeared at 5.50 and 5.72 ppm and a peak corresponding to the methyl group (CH_2_=C(**CH**_**3**_)-CONH−), observed at 1.98 ppm, of the methacryloyl functionalities on the modified gelatin (Gel-MA); characteristic peaks at 6.00 and 6.83 ppm assigned to two methine (CH_3_-**CH=CH**-CONH−) protons and a peak at 1.91 ppm attributed to the methyl (**CH**_**3**_-CH=CH-CONH−) group of the crotonoyl groups (Gel-CA); new proton peaks belonging to the methylidene (**CH**_**2**_=C(COOH)-CH_2_-CONH−) group identified at 5.54 and 6.01 ppm and a peak at 3.21 ppm assigned to the methylene (CH_2_=C(COOH)-**CH**_**2**_-CONH−) group of the itaconoyl groups (Gel-IA). All spectra of modified gelatin samples confirm successful modification with each anhydride at all molar ratios. ^1^H NMR spectra of native gelatin and its derivatives with different molar ratios as well as the anhydrides are illustrated in Figures S3–S7 in the Supporting Information. In the case of Gel-IA, a decrease of the lysine methylene signal at 3.06 ppm with an increasing amount of IA in the reaction confirmed the modification of lysine residues on the gelatin backbone, whereas in the reaction with CA and MA at all molar ratios, the lysine signals disappeared, indicating the complete conversion of the amino groups. This can most likely be attributed to the increased reactivity of CA and MA compared to IA. Since the reaction takes place in an aqueous environment, the anhydrides will react, however, the crotonic and methacrylic counterparts will do so more quickly, leading to a higher reagent consumption. Moreover, reactions with MA resulted in derivatives, whose spectra display additional small peaks at 6.17 ppm, suggesting that in addition to reacting with lysine residues, some methacryloyl moieties are introduced through the reactions with other side groups in gelatin, e.g., hydroxyl groups, due to the increased reaction time. Table 1 summarizes the data on the degree of functionalization (DoF) of gelatin based on the molar ratios determined via both quantitative ^1^H NMR analysis and TNBSA assay as well as the product yields.

**Table 1:** Modification of Gelatin with Different Anhydrides and Resulting DoF Determined Using ^1^H NMR Spectroscopy and a TNBSA Assay, as Well as Other Physicochemical Characteristics^a^

sample^b^	*x*-fold molar excess of an anhydride	DoF by ^1^H NMR (%)	DoF by TNBSA assay (mmol/g)^c^	yield (%)	IEP_EM_	IEP_viscometry_
gelatin			d		7.0	∼6.0
Gel-MA_1.5_	1.5	117 ± 1	0.373	91 ± 1	4.2	4.2
Gel-MA_3_	3	122 ± 2	0.380	88 ± 2	4.2	4.1
Gel-MA_6_	6	132 ± 2	0.384	86 ± 3	4.3	4.4
Gel-CA_1.5_	1.5	121 ± 2	0.355	85 ± 2	2.5	ND
Gel-CA_3_	3	126 ± 1	0.358	79 ± 2	3.2	ND
Gel-CA_6_	6	133 ± 2	0.360	68 ± 1	3.2	ND
Gel-IA_2_	2	67 ± 1	0.274	86 ± 1	3.8	4.0
Gel-IA_5_	5	79 ± 3	0.298	78 ± 2	3.8	4.0
Gel-IA_10_	10	79 ± 2	0.298	65 ± 1	3.5	ND

a Gel-MA, Gel-CA, and Gel-IA are methacryloylated, crotonoylated, and itaconoylated gelatins, respectively; DoF, degree of functionalization; TNBSA assay, 2,4,6-trinitrobenzenesulfonic acid assay; IEP, isoelectric point; ND, not detectable.

b The suffixes denote the molar excess of anhydrides added referring to the amount of free amino groups in gelatin.

c The amount of incorporated unsaturated groups. Results are given as the mean ± standard deviation values (*n* = 3).

d The total amount of free amino groups in a native gelatin was determined to be 0.434 mmol/g.

The determination of DoF values was also done using a glycine standard curve through TNBSA assay (see Figure S1 in the Supporting Information). The total amount of free amino groups in pristine gelatin was found to be 0.434 mmol/g, which is in good agreement with the data published earlier.^40,55^ The amount of the remaining free amines after gelatin derivatization was then subtracted from the amount of free −NH_2_ groups in a native gelatin to calculate the amount of introduced unsaturated groups (Table 1).

The derivatization of gelatin was further confirmed using FTIR spectroscopy (see Figure S8 in the Supporting Information). FTIR analysis of spectra clearly showed the key absorbance peaks: the spectrum of the parent gelatin shows the presence of the broad band (3600–3100 cm^–1^) with a maximum peak at 3280 cm^–1^ and a shoulder at 3067 cm^–1^ representing the asymmetric and symmetric N–H stretching vibrations (amide A), respectively, which overlaps with −OH stretching in the same region. The peaks at 2935 and 2872 cm^–1^ correspond to the asymmetric and symmetric stretching vibrations in CH_2_ groups. The characteristic absorption bands at 1630, 1523, and 1234 cm^–1^ are assigned to the C=O stretching (amide I), N–H bending plus C–H stretching (amide II), and C–N stretching coupled to N–H bending (amide III) vibrations, respectively, and are in good agreement with the FTIR data on gelatin reported in the literature.^56−60^ The peaks at 1439 and 1332 cm^–1^ are due to CH bending and the peak at 1078 cm^–1^ represents C–C stretching. The introduction of crotonoyl, itaconoyl, and methacryloyl moieties caused small shifts in the amide bands of gelatin to higher frequencies. An absorption band typical for vinyl groups (C=C stretching vibration) should be visible at 1680–1620 cm^–1^, however, in our case, this was overlapped with the amide I signal (see Figure S8 in the Supporting Information). Nevertheless, the intensities of the amide I, II, and III peaks increased, which can be attributed to the incorporated amide bonds coupled with a C=C stretching vibration. Overall, similar infrared spectra were acquired for different batches of modified gelatins, suggesting the limitations of FTIR spectroscopy in confirming the effectiveness of gelatin derivatization when preparing various batches.

The amphoteric nature of gelatin is due to the presence of amino and carboxylic groups present in amino acids in the macromolecular chains. At pHs lower than isoelectric point (IEP), the macromolecules of gelatin carry a positive charge; whereas at pH > IEP they are negatively charged. At pH = IEP gelatin has a net charge of zero.^61^ The IEP also represents the point at which the polyampholyte chains adopt their most compact conformation resulting in minimal viscosity in solutions.^62,63^

**Figure 2:**
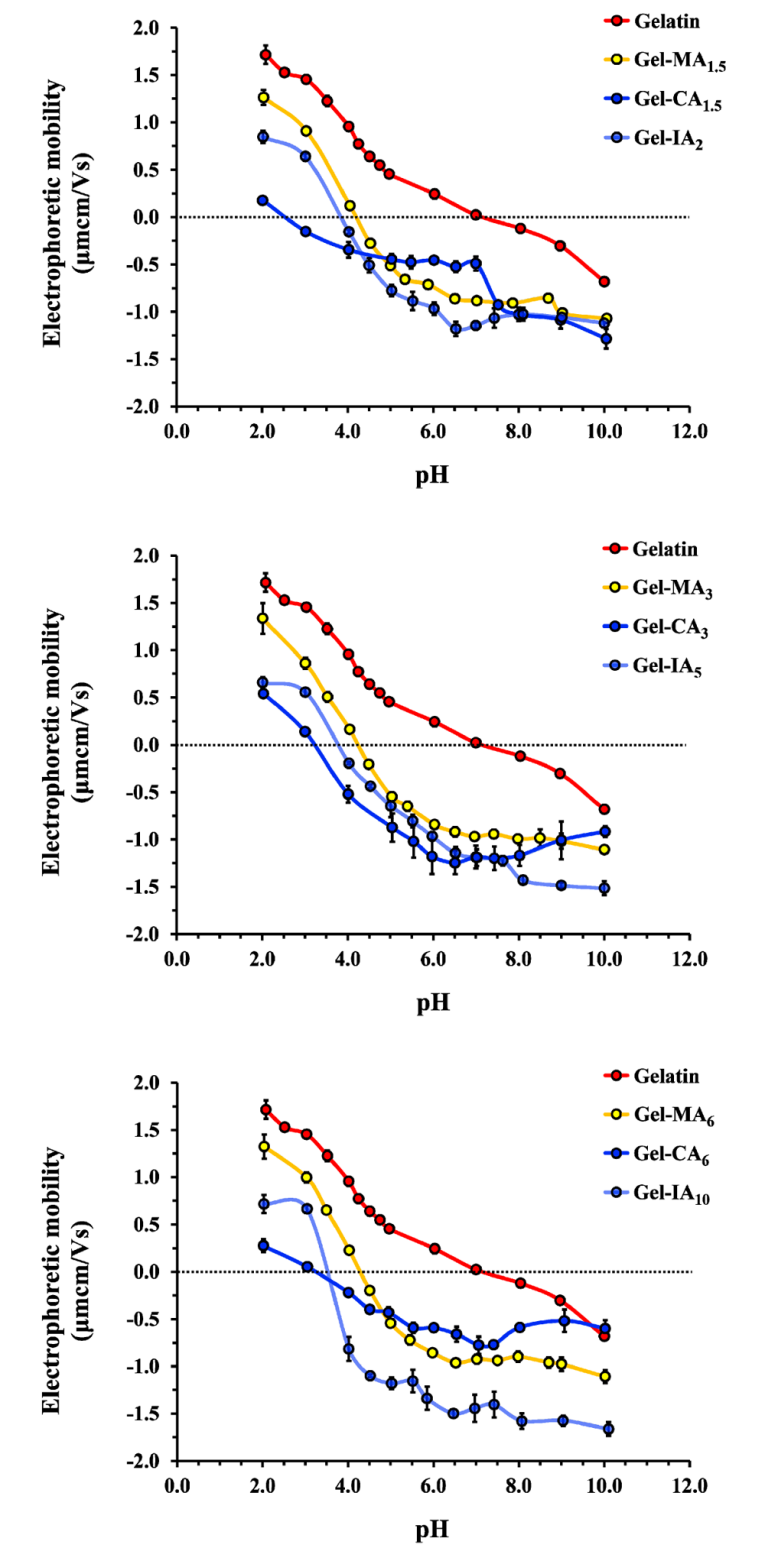
Electrophoretic mobility as a function of solution pH curves, recorded to determine the IEP_EM_ of native gelatin and its chemically modified derivatives (Gel-MA, Gel-CA, and Gel-IA are methacryloylated, crotonoylated, and itaconoylated gelatins, respectively).

In this study the IEPs for both derivatized and underivatized gelatin macromolecules were determined using the measurements of the electrophoretic mobility and following the changes in solution viscosity as a function of pH. Aqueous solutions (1% w/v) of gelatin and its modified derivatives were titrated by adding varying amounts of acid (0.03 M HCl) or base (0.02 M NaOH), and resulting pH changes were followed using a pH meter. The IEPs of the samples were estimated by determining the pH value at which the electrophoretic mobility curve crossed zero (Figure 2) or when the polyampholyte solution exhibited a minimum specific viscosity (see Figure S9 in the Supporting Information). It was found that native gelatin (type A) produced from porcine skin has an IEP_EM_ of pH 7.0, which is within the range given by the manufacturer. It was expected that the incorporation of unsaturated anhydride groups into lysine moiety of gelatin would lower the isoelectric point significantly. Indeed, the introduction of CA, IA and MA groups, and the loss of the lysine primary amines, resulted in a reduction of the IEP of the modified gelatin derivatives below that of native gelatin indicating the successful chemical modification of this biopolymer (Table 1). The observed changes in IEP during gelatin derivatization are evidently influenced by two factors: the degree of gelatin functionalization and the nature of the introduced functional groups. The incorporation of itaconoyl groups into gelatin leads to a more pronounced reduction in IEP. This effect is attributed to the presence of an additional carboxylic acid group within the itaconoyl moiety.

Gelatin solutions in water are known to exhibit sol-to-gel transitions upon decrease in temperature. This behavior for native gelatin and its new derivatives was studied using rheological measurements. The rheological changes happening with 5% (w/v) aqueous solutions of native gelatin and its chemically modified derivatives (Gel-CA, Gel-IA, and Gel-MA) upon changes in temperature are displayed in Figures S10 and S11 in the Supporting Information. Considering the trend of the data obtained, both the melting (*T*_m_) and gelation (*T*_gel_) points can be determined by taking a crossover temperature between the storage modulus (*G*′) and loss modulus (*G*′′).^40,64,65^ The crossover temperature is attributed to the “gel-to-sol” or “sol-to-gel” transition temperature, which indicates the transition from an elastic network formation to a solution upon heating or a physically cross-linked gel formation, respectively. It was found that the native gelatin (type A, from porcine skin) exhibited a *T*_m_ at 30.4 ± 0.3 °C and a *T*_gel_ at 17.2 ± 1.2 °C during heating and cooling cycles, respectively. The obtained data on melting and gelation temperatures of native gelatin are in good agreement with the data reported in the literature.^64,66^ Gelation and melting temperatures of gelatin derivatives were observed to have a good correlation with their degree of functionalization (DoF). The lower *T*_gel_ and *T*_m_ points were detected for gelatin derivatives with the greater DoF, indicating that chemical modification affects the gelling properties of gelatin. The melting and gelation temperatures of these biopolymers are presented in Table 2.

**Table 2:** Gelation (*T*_gel_) and Melting (*T*_m_) Temperatures of Derivatized and Underivatized Gelatins Determined Using Dynamic Rheological Measurements, where a Crossover Temperature of Storage Modulus (*G*′) and Loss Modulus (*G*′′) Occurs upon Cooling and Heating Scans, Respectively^a^

sample	*T*_gel_ (°C)	*T*_m_ (°C)
gelatin	17.2 ± 1.2	30.4 ± 0.3
Gel-MA_1.5_	11.5 ± 0.8	27.1 ± 0.6
Gel-MA_3_	10.8 ± 1.0	23.3 ± 1.3
Gel-MA_6_	8.3 ± 0.6	21.8 ± 0.6
Gel-CA_1.5_	12.4 ± 1.4	27.3 ± 0.8
Gel-CA_3_	7.6 ± 1.4	25.1 ± 1.2
Gel-CA_6_	5.7 ± 1.2	23.9 ± 1.7
Gel-IA_2_	15.6 ± 1.1	27.6 ± 0.9
Gel-IA_5_	14.1 ± 1.0	26.5 ± 0.5
Gel-IA_10_	12.7 ± 1.3	26.0 ± 1.3

a Gel-MA, Gel-CA, and Gel-IA are methacryloylated, crotonoylated, and itaconoylated gelatins, respectively.

### 3.2 Toxicology

#### 3.2.1 Cell Viability

**Figure 3:**
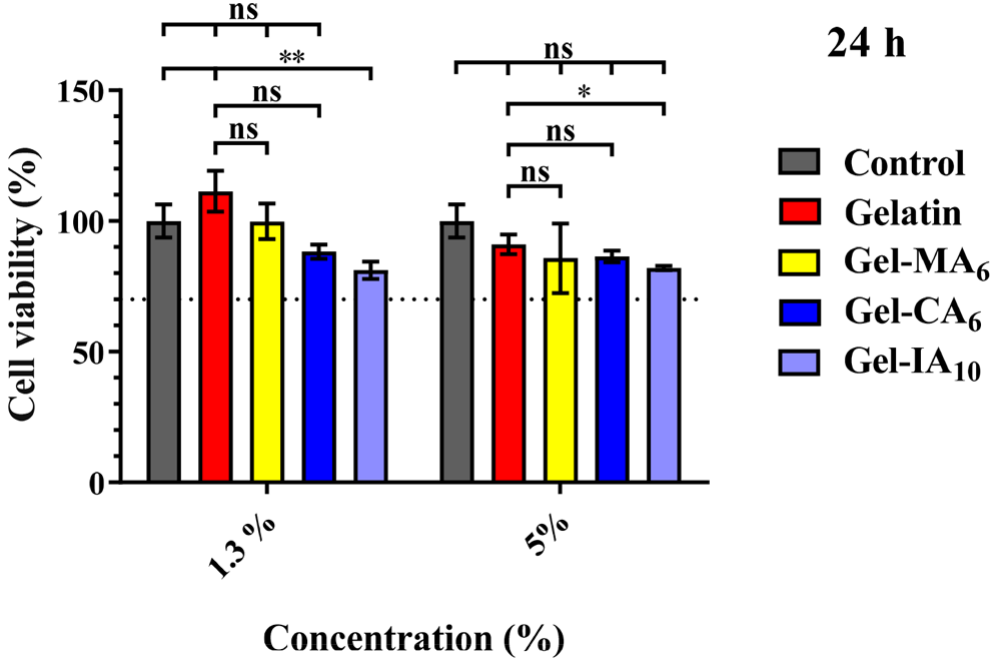
Effect of modified and unmodified gelatin formulations at 1.3 and 5% (w/v) concentrations on the percentage of viable HPF cells after 24 h exposure assessed using MTT assay. Data represented as the mean ± standard error of the mean of two independent experiments in quadruplicate. Gel-MA_6_, Gel-CA_6_, and Gel-IA_10_ are methacryloylated, crotonoylated, and itaconoylated gelatins, respectively. Statistically significant differences are shown as *p < 0.05; **p < 0.01; “ns” denotes no significance.

*In vitro* cytotoxicity of gelatin and its modified derivatives (Gel-CA, Gel-IA, and Gel-MA) was studied using MTT assay with human pulmonary fibroblasts (HPF) cells. The assay is based on the ability of mitochondria of live cells to reduce 3-(4,5-dimethylthiazol-2-yl)-2,5-diphenyltetrazolium bromide (MTT reagent), a yellow substance, to insoluble formazan crystals (violet color). This technique allows to calculate the number of viable cells after treatment with the test material. HPF cells were treated with gelatin, Gel-CA_6_, Gel-IA_10_, and Gel-MA_6_ solutions at concentrations of 1.3 and 5% (w/v) in cell growth media for 24 h. The negative control group consisted of untreated cells was considered as 100% of viable cells. Figure 3 displays the data on cell viability in the presence of modified and unmodified gelatins. MTT results showed that cell viabilities are comparable for gelatin and its chemically modified derivatives as there were no statistically significant differences in viability between these biopolymers (*p* > 0.05) against the control after 24 h of treatment in the studied concentrations. Among the considered, only Gel-IA_10_ exhibited a slight decrease in cell viability at 1.3% concentration to 81.1 ± 9.4%. However, according to the ISO standards developed by the United Nations Sustainable Development Goals (SDGs; ISO 10993-5:2009) the material for biomedical application is considered safe as long as the *in vitro* viability is 70% and above.^67^ Our results show that the level of cell viability remained high (>70%) after the treatment with all biopolymers at given concentrations (Figure 3).

Since gelatin is considered as a safe material,^68,69^ in the next step, we compared the viability of gelatin-treated cells with the group treated with its modified derivatives. Gel-MA and Gel-CA as well as pure gelatin did not show a significant difference in viability (*p* > 0.05). Moreover, it can be seen that the viability of gelatin was higher than that of the control group. It can be assumed that pure gelatin at a lower concentration shows some ability to facilitate cell proliferation. Our results are in good agreement with previous reports,^70−72^ and gelatin and its modified derivatives can be considered safe in this regard.

In the majority of cases, the difference between gelatin and its modified derivatives was not statistically significant (*p* > 0.05), which indicates that chemical modification of gelatin with unsaturated groups does not cause an increase in the polymer toxicity. It can be concluded that the synthesized Gel-CA, Gel-IA, and Gel-MA are nontoxic and suitable for further development in pharmaceutical applications.

#### 3.2.2 Mucosal Irritancy

The *in vivo* slug mucosal irritation test (SMIT) was originally developed by Adriaens and co-workers^73,74^ and has been validated as an alternative and reliable method for evaluating the mucosal irritation potency of various chemicals, excipients, cosmetics, formulations, and active ingredients. This technique has been applied in many studies, including the evaluation of nasal and vaginal irritation.^52,75−83^ SMIT uses terrestrial slugs, which are not protected by legislation controlling animal experiments and are considered to have limited sentience.^73,84^ Mucus secretion is essential for slugs to aid their locomotion and prevent dehydration. They also release mucus and lose body weight when in contact with irritants. When their mucosal membrane is damaged the slugs produce additional proteins and enzymes. The test provides quantifiable end points for classifying test materials into nonirritating (MP% ≤ 5.5), mild (MP% 5.5–10), moderate (MP% 10–17.5), or severely irritating (MP% ≥ 17.5) based on the levels of mucus production. In general, mild irritants cause an increase in mucus production; however, strong irritants induce tissue erosion in addition to increased mucus production.^81,82^

**Figure 4:**
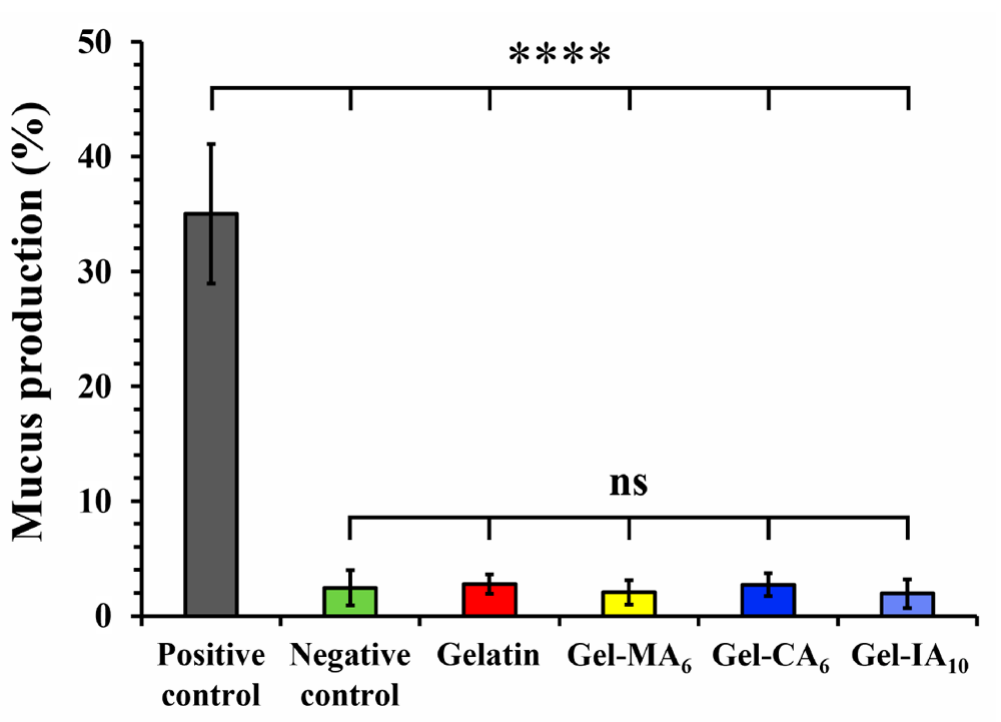
Mucus production (MP%) by *Arion lusitanicus* slugs in response to 60 min contact with gelatin and its methacryloylated (Gel-MA_6_), crotonoylated (Gel-CA_6_), and itaconoylated (Gel-IA_10_) derivatives, as well as positive (benzalkonium chloride; BAC) and negative (phosphate buffered saline; PBS) controls. Data are expressed as mean ± standard deviation values (*n* = 7). Statistically significant differences are given as ****p < 0.0001; “ns” denotes no significance.

The modified version of this assay is routinely used by our research group for assessing the biocompatibility of different polymeric excipients.^23,24,49,85^Figure 4 presents the data on the percentage of mucus production (MP%) by *Arion lusitanicus* slugs after 60 min of exposure to a filter paper soaked with solutions of gelatin and its modified derivatives as well as positive and negative controls. All test materials used in this assay were dissolved in phosphate-buffered saline (PBS; pH 7.40). Gelatin-based samples were prepared at 1.3% (w/v), as at this concentration these samples were able to form a thin gel layer on top of the filter paper. Slugs placed in 1% (w/v) BAC solution (positive control; pH 7.36) exhibited an extreme discomfort, producing significantly larger amounts of yellow up to orange-colored mucus (35 ± 6%) than those exposed to PBS solution (negative control; pH 7.40) with a very low level of MP% (2 ± 1%; *p* < 0.0001). These results are in good agreement with the previous studies.^23,24,49,73^ Mucus production values recorded for the slugs exposed to gelatin, Gel-MA_6_, Gel-CA_6_, and Gel-IA_10_ (pH 7.32–7.41) were 3 ± 1, 2 ± 1, 3 ± 1, and 2 ± 1%, respectively. The mucus secretions were colorless, which serves as a good initial indicator of biocompatibility.^24^ No statistically significant differences (*p* ≫ 0.05) in MP% were observed between the values recorded for the negative control and gelatin-based test materials, demonstrating the nonirritating nature of both gelatin and modified gelatins. The results provide valuable insights into the biocompatibility of gelatin derivatives and suggest that they could be potentially used in various applications without causing mucosal irritation. Figures S12 and S13 in the Supporting Information provide a detailed schematic illustration of the SMIT assay procedure and photographs with *Arion lusitanicus* slugs exposed to the test materials, respectively.

### 3.3 Mucoadhesion Studies

#### 3.3.1 Retention on *Ex Vivo* Porcine Vaginal Tissues

The vaginal route of drug administration is often preferred for the local treatment of various gynecological dysfunctions and infections. Local administration has the potential to improve drug absorption and delivery to target tissues while reducing adverse effects. However, vaginal drug administration requires overcoming several obstacles to achieve effective drug absorption and retention. The vaginal epithelium that consists of multiple layers of cells tightly packed together limits the penetration of drugs. Moreover, the presence of a thick layer of mucus within the vaginal cavity further hinders drug diffusion into the underlying tissues. The mucus layer, composed of glycoproteins and mucins, serves as a viscoelastic protective barrier, clearing and lubricating the reproductive tract epithelia to help eliminating pathogens and foreign substances. Furthermore, the physicochemical properties of vaginal fluid, including its volume, viscosity, and acidic pH of the vaginal environment may also have an unfavorable impact on drug absorption and retention. Additionally, such factors as vaginal physiology, age, menstrual cycle, reproductive system disorders, and formulation parameters can also affect the rate and extent of drug absorption in the vaginal cavity.^86−88^ Collectively, these anatomical factors pose challenges in achieving effective drug delivery when administered intravaginally.

In this study, the potential use of gelatin and its crotonoylated, itaconoylated, and methacryloylated derivatives in vaginal drug delivery was studied using an *in vitro* assay based on flow-through with fluorescent detection. The formulations were prepared with fluorescein sodium (NaFl), which is a fluorescent marker that facilitates easy detection and measurement of mucosal retention levels. This method has been widely employed to study the retention of various formulations on different mucosal surfaces, including vaginal tissues.^10^ The effectiveness of this technique was validated against other established methodologies used to assess mucoadhesive properties.^89^

**Figure 5:**
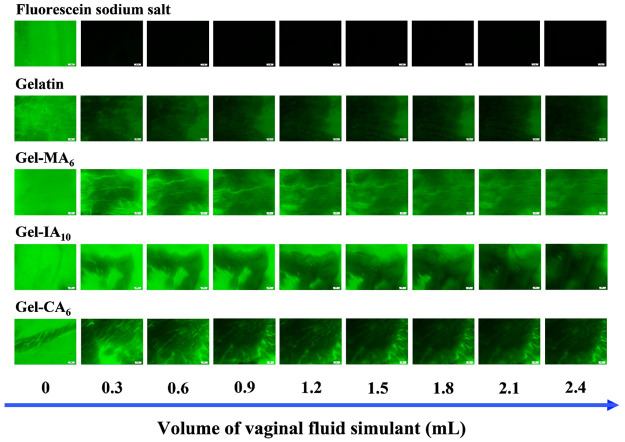
Selected fluorescence images showing mucosal retention of 5% w/v native gelatin, methacryloylated gelatin (Gel-MA_6_), itaconoylated gelatin (Gel-IA_10_), and crotonoylated gelatin (Gel-CA_6_) formulations containing 0.1 mg/mL fluorescein sodium (NaFl), as well as free 0.1 mg/mL NaFl (used as a nonmucoadhesive control), on freshly dissected porcine vaginal tissue after washing with varying volumes of VFS solution (pH 4.0; flow rate 300 μL/min). Fluorescence microscope parameters: magnification −1.25×; exposure time −10 ms; gain −1.0×. Scale bars correspond to 2 mm.

**Figure 6:**
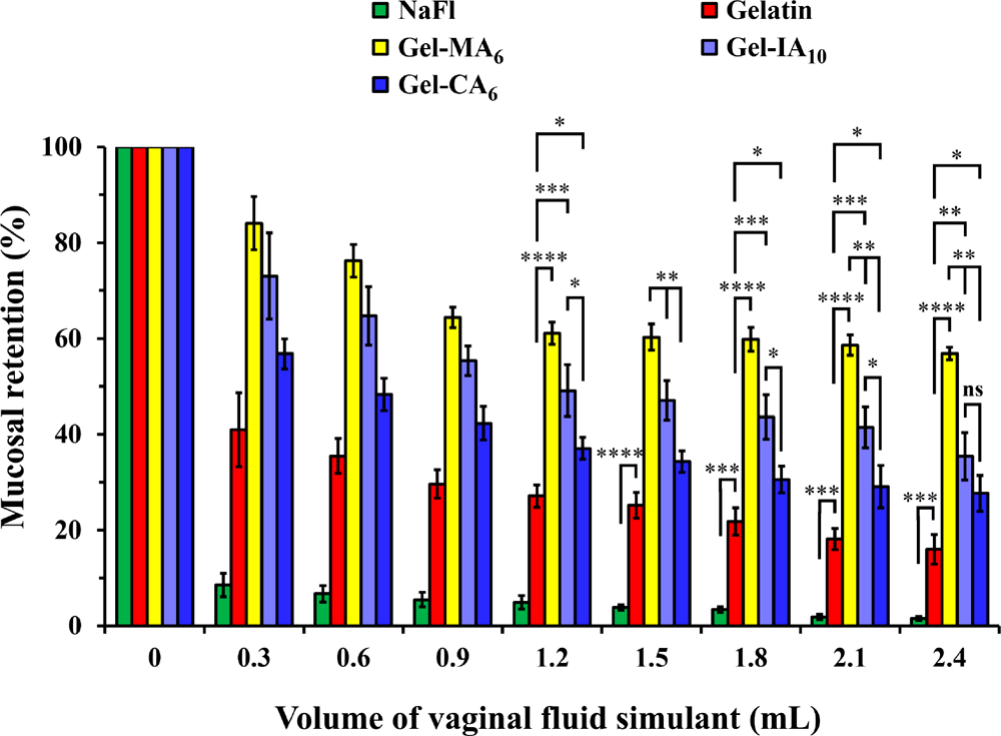
Percentage retention of 5% w/v native gelatin, methacryloylated gelatin (Gel-MA_6_), itaconoylated gelatin (Gel-IA_10_), and crotonoylated gelatin (Gel-CA_6_) formulations containing 0.1 mg/mL fluorescein sodium (NaFl) as well as free 0.1 mg/mL NaFl (used as a nonmucoadhesive control) on freshly excised porcine vaginal mucosa after irrigating with different volumes of VFS solution (pH 4.0; flow rate 300 μL/min). Data are expressed as mean ± standard deviation values (*n* = 3). Statistically significant differences are represented as *p < 0.05; **p < 0.01; ***p < 0.001; ****p < 0.0001; “ns” denotes no significance.

Briefly, NaFl-containing gelatin and its chemically modified derivatives (Gel-CA_6_, Gel-IA_10_, and Gel-MA_6_), as well as free NaFl solution, were administered on the surface of *ex vivo* porcine vaginal mucosa and allowed to equilibrate at 37 °C. The mucosal surface was then irrigated with varying volumes of vaginal fluid simulant (VFS; pH 4.0; flow rate 300 μL/min), and the presence of the formulation on the mucosal surface was determined using a fluorescence microscope. The total volume of VFS used in this wash-off experiment was aimed to mimic the amount of normal vaginal discharge in healthy women (∼1–3 mL daily).^90^ The exemplar fluorescent microphotographs of the retention of these formulations taken after each wash with VFS are illustrated in Figure 5. The mucosal retention can be quantified by measuring the change in image pixel intensity over time to give a percentage of the fluorescence relative to the initial time (Figure 6). Image analysis helped to reveal that the incorporation of crotonoyl, itaconoyl, and methacryloyl functional groups into gelatin structure significantly enhanced formulation retention on freshly excised porcine vaginal mucosa. Among modified gelatins, Gel-MA_6_ demonstrated superior mucoadhesive performance compared to native gelatin (*p* < 0.0001) and fluorescein sodium solution (*p* < 0.0001) throughout the wash-off experiment. As anticipated, the polymer-free solutions of NaFl (served as a nonmucoadhesive control) exhibited significantly poorer retention and was rapidly washed out from the mucosal surface, with only ∼1.6% fluorescence observed upon completion of the full washing cycle. Traditionally, native gelatin is considered as a polymer with poor retention capabilities and the results generated during the *ex vivo* mucoadhesion experiments corroborated this assessment. Thus, pure gelatin displayed considerably weaker adhesion to the vaginal mucosa in comparison with its modified counterparts: Gel-MA_6_ (*p* < 0.0001); Gel-IA_10_ (*p* < 0.001); and Gel-CA_6_ (*p* < 0.05), however, a greater retention compared to NaFl solution (*p* < 0.001). It was observed that Gel-IA_10_ exhibited a small but significantly greater retention (*p* < 0.05) on vaginal mucosa in contrast to Gel-CA_6_ during the mucoadhesion experiment. However, no statistically significant difference (*p* > 0.05) was found between these formulations at the end of the wash-off test (Figure 6). It is worth noting that Gel-MA_6_ displayed better mucoadhesive performance (*p* < 0.01) compared to the other modified gelatins (Gel-CA_6_ and Gel-IA_10_). Previously, we have reported that polymers modified with methacryloyl groups exhibited substantially improved adhesion to various mucosal surfaces and demonstrated comparable or even better mucoadhesive performance at higher degrees of functionalization relative to chitosan, which is commonly regarded as a “gold standard” mucoadhesive polymer/positive control in this field.^10,91^

**Figure 7:**
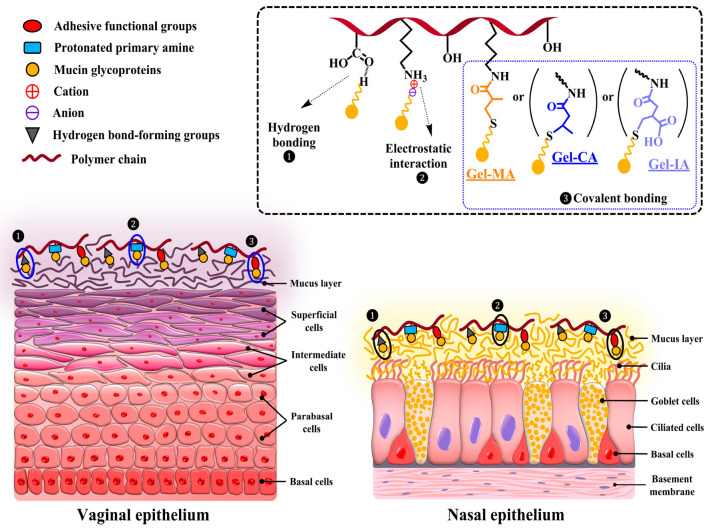
Schematic illustration of the retention of methacryloylated gelatin (Gel-MA), crotonoylated gelatin (Gel-CA), and itaconoylated gelatin (Gel-IA) formulations on vaginal and nasal mucosal surfaces.

As expected, retention of all formulations declines over the course of the washing, yet the following trend is observed: Gel-MA_6_ > Gel-IA_10_ > Gel-CA_6_ > gelatin ≫ NaFl. According to these findings, it is reasonable to assume that the excellent mucoadhesive performance of these formulations is due to their interaction with mucosal surfaces via three mechanisms, as illustrated in Figure 7: (i) the ability of unsaturated functional groups (methacryloyl, crotonoyl, itaconoyl) of modified gelatins to form covalent bonds with thiol groups present in the mucus layer through Michael-type addition reaction; (ii) electrostatic interaction between residual protonated primary amino groups within the modified gelatins and negatively charged mucins present on mucosal surface; and (iii) hydrogen bonding between hydroxyl, carboxyl groups of gelatin and mucin glycoprotein moieties.

The electrostatic interaction contribution of amphoteric macromolecules to mucoadhesion is highly dependent on the solution pH and its relative position against the IEP of polyampholyte.^92^ This, in turn, relies on the degree of gelatin functionalization and the nature of the introduced functional groups. Considering that the retention experiments were conducted in VFS at pH 4.0, only unmodified gelatin will carry a strong positive charge under these conditions, given its IEP at 7.0. Gelatin derivatives with methacryloyl groups exhibit IEP values between 4.2–4.3, resulting in only weak positive charges. Derivatives with crotonoyl and itaconoyl groups possess IEP values ranging from 2.5–3.2 and 3.5–3.8, respectively, making these polymers negatively charged during the retention experiments. Consequently, it is reasonable to infer that electrostatic binding of gelatin derivatives to the vaginal surface is not the primary factor contributing to mucoadhesion.

#### 3.3.2 Retention on *Ex Vivo* Sheep Nasal Tissues

Intranasal administration offers a noninvasive route of drug delivery. Therapeutic agents delivered to the nasal cavity act locally and provide a direct target to the central nervous system. Estimated daily production of nasal mucus varies between 0.1 and 0.3 mg/kg (or from 20 to 40 mL) under normal conditions.^93,94^ However, the exact amount of mucus secretion can be influenced by various factors such as environmental conditions, allergies, infections, individual variations, as well as specific location and region of the human nasal mucosa. Mucociliary clearance is a complex and dynamic physiological process that helps maintaining the upper and lower airways clean and defend against airborne particles and pathogens. This is done through the interaction of nasal mucus and ciliary beating. The coordinated waves of tiny hair-like cilia gradually move the thick mucus from the front of the nose to the nasopharynx, where it can either be swallowed or expectorated. Additionally, the nasal mucus is slightly acidic (pH 5.5–6.0) in order to prevent respiratory infections.^93,95^ The protective mechanism of the respiratory system functions efficiently and can greatly limit the residence time of therapeutic substances when administered via the nasal route. Mucoadhesive dosage forms are designed to counteract the clearance mechanism by adhering to the mucosal surface, therefore prolonging the retention time and improving effective drug absorption.

Microparticulate formulations are commonly used in nasal drug delivery.^96^ Therefore, in this study, gelatin and its derivatives were formulated as microparticles with NaFl as a model drug. Two types of microparticles were designed with and without the use of a cross-linking agent to evaluate the role of gelatin cross-linking on their nasal retention properties. NaFl-loaded cross-linked and non-cross-linked microparticles based on gelatin and its modified derivatives (Gel-CA; Gel-IA and Gel-MA) have been successfully produced using a spray drying technique. The difference between cross-linked and non-cross-linked particles was the addition of glutaraldehyde, which facilitates cross-linking of gelatin macromolecules. The surface morphology of these microparticles was characterized using scanning electron microscopy (SEM). Microparticles collected after spray drying were spherical and presented a wrinkled surface texture, free of crystals, pores, and cracks (see Figures S14 and S15 in the Supporting Information). All the formulations led to microparticles with similar morphologies and the mean diameters of the particles were ∼5 ± 1 μm, which were within the range expected for microparticles generated by common spray drying techniques.^97,98^

**Figure 8:**
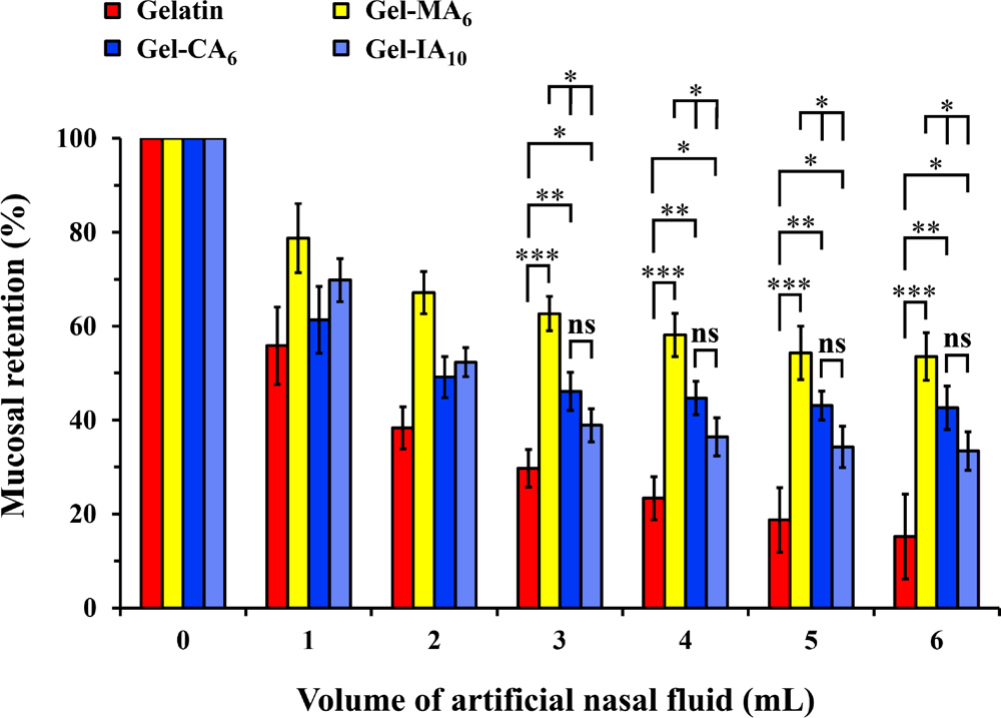
Percentage retention of ∼100 mg of pristine gelatin, methacryloylated gelatin (Gel-MA_6_), crotonoylated gelatin (Gel-CA_6_), and itaconoylated gelatin (Gel-IA_10_) non-cross-linked microparticle formulations, each containing 1 mg/mL fluorescein sodium (NaFl), on freshly dissected sheep nasal mucosa. The mucosa was washed with varying volumes of ANF solution (pH 5.80; flow rate 200 μL/min). Data are expressed as mean ± standard deviation values (*n* = 3). Statistically significant differences are given as *p < 0.05; **p < 0.01; ***p < 0.001; “ns” denotes no significance.

In this experiment, we have evaluated the potential of modified and unmodified gelatin-based spray-dried microparticles as mucoadhesive formulations for their application in nasal drug delivery. The retention properties of these formulations were studied involving the same *in vitro* flow-through method as described above with some modifications. Only non-cross-linked microparticle samples were used in this experiment. The purpose of using non-cross-linked microparticles is to facilitate their adhesion by allowing them to slowly swell and dissolve upon contact with wet mucosal surface when applied/inhaled. Briefly, ∼100 mg of modified and unmodified gelatin-based microparticle samples containing NaFl were deposited on *ex vivo* sheep nasal mucosa. The experiment was conducted at 37 °C in an incubator. In the course of this experiment, ANF solution flowing down the nasal mucosa during each washing cycle was collected at predetermined time points. Subsequently, these samples were analyzed using a fluorescence spectrophotometer. Using the data acquired and generating a standard curve for microparticles with NaFl, the amount of dosage form washed off the nasal mucosa could be calculated. Consequently, through reverse calculations, the amount of retained formulations on sheep nasal mucosa could be estimated (Figure 8). All samples exhibited a reduction in determined fluorescence upon washing process, indicating the removal of gelatin-based microparticle formulations from the mucosal surface. However, methacryloylated gelatin (Gel-MA_6_) microparticles demonstrated stronger adhesion (*p* < 0.05) to nasal mucosa compared to other modified gelatin derivatives (Gel-CA_6_ and Gel-IA_10_). Once again, pristine gelatin showed poorer mucoadhesive performance in contrast to its modified counterparts: Gel-MA_6_ (*p* < 0.001); Gel-CA_6_ (*p* < 0.01); and Gel-IA_10_ (*p* < 0.05). Interestingly, no statistically significant difference (*p* > 0.05) was observed between Gel-CA_6_ and Gel-IA_10_ formulations throughout the experimental process, as they displayed a similar retention profile. Overall, these observations are consistent with the previous results and indicate that the three biopolymer derivatives examined have a greater retention on nasal mucosa than native gelatin due to their enhanced mucoadhesive properties.

To further corroborate the findings obtained from the *in vitro* flow-through method, a tensile test was employed in order to assess the adhesion of microparticles based on cross-linked and non-cross-linked gelatin and its chemically modified derivatives to freshly excised sheep nasal mucosa. The tensile method is one of the widely used and established techniques to evaluate the mucoadhesive properties of various dosage forms.^10^ This method involves the bringing polymeric mucoadhesives in contact with mucosal tissue with its subsequent withdrawal, followed by recording and analysis of the resulting detachment profiles. The mucoadhesive performance of dosage forms is then determined through a combination of two important parameters: the measurement of maximal force of detachment required to separate the dosage form from mucosa; and the total work of adhesion, defined as the area under the corresponding detachment force versus distance curve (see Figures S16 and S17 in the Supporting Information for the exemplar detachment profiles of test materials).

**Figure 9:**
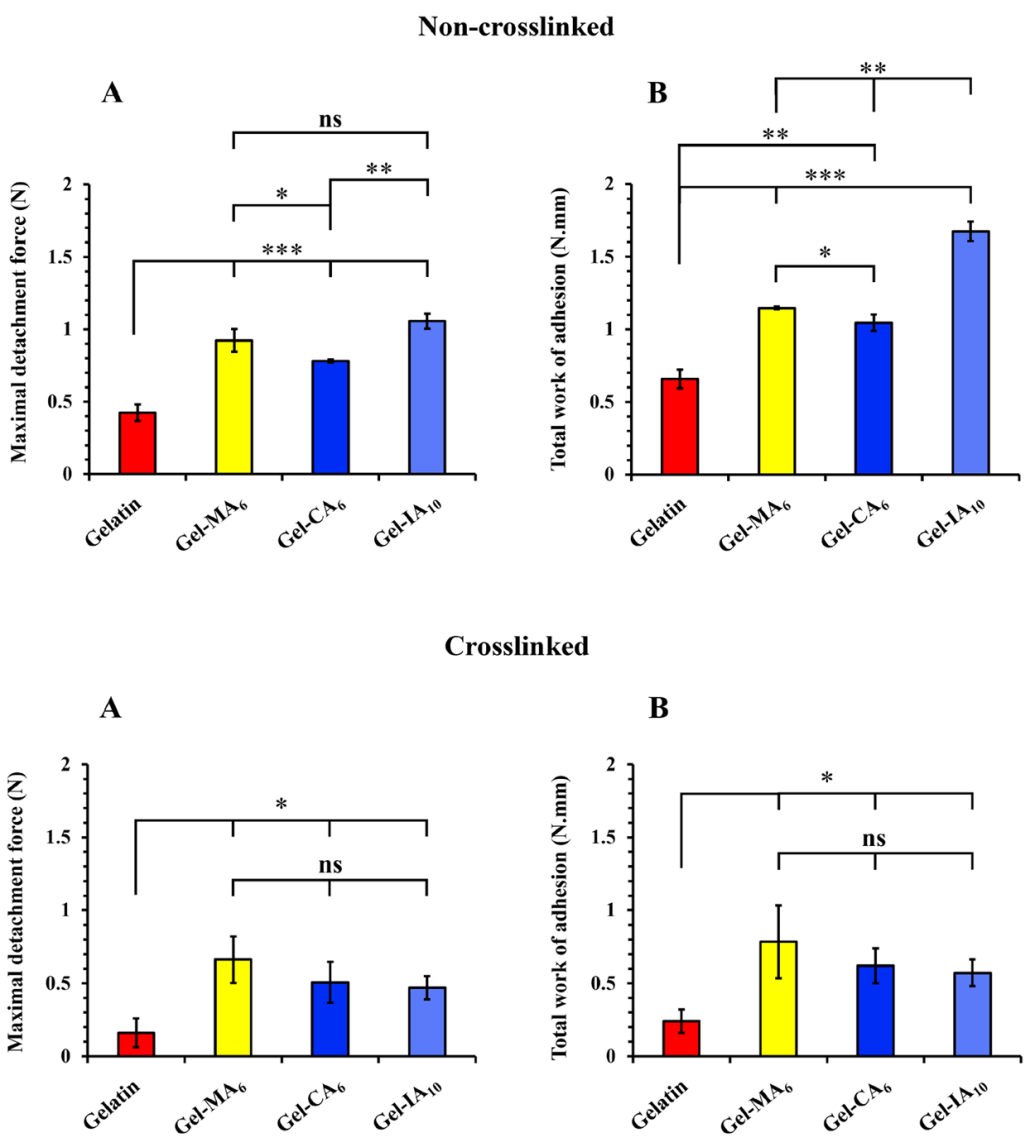
Mucoadhesive characteristics of ∼100 mg of different biopolymers: (A) Maximal force of detachment and (B) total work of adhesion profiles for microparticles based on cross-linked and non-cross-linked gelatins and their chemically modified derivatives determined by texture analysis. Data are expressed as mean ± standard deviation values (*n* = 3). Statistically significant differences are displayed as *p < 0.05; **p < 0.01; ***p < 0.001; “ns” represents no significance.

Figure 9 illustrates the values of maximal force of detachment (*F*_det_) and total work of adhesion (*W*_adh_), as calculated from tensile test studies for both cross-linked and non-cross-linked gelatin-based microparticles. Chemically modified gelatin (Gel-MA_6_, Gel-CA_6_, and Gel-IA_10_) formulations exhibited superior mucoadhesive performance (*p* < 0.001) expressing higher *F*_det_ and *W*_adh_ profiles when compared to parent gelatin within non-cross-linked microparticles. It was revealed that samples based on non-cross-linked Gel-CA_6_ microparticles displayed the least retention performance compared to Gel-MA_6_ (*p* < 0.05) and Gel-IA_10_ (*p* < 0.01) counterparts showing a lower *F*_det_ value. No statistically significant difference was observed in *F*_det_ profiles between Gel-MA_6_ and Gel-IA_10_, however, Gel-IA_10_ (*p* < 0.01) exhibited a higher *W*_adh_ value in contrast to Gel-MA_6_ and Gel-CA_6_.

Interestingly, there were no statistically significant differences observed between the cross-linked modified gelatin (Gel-MA_6_, Gel-CA_6_, and Gel-IA_10_) microparticles, as they exhibited very similar *F*_det_ and *W*_adh_ profiles, indicating comparable mucoadhesive performance among these type of dosage forms. Overall, pristine gelatin displayed the poorest mucoadhesive properties in comparison to its modified counterparts within both cross-linked and non-cross-linked microparticles.

The introduction of specific unsaturated crotonoyl, itaconoyl and methacryloyl functional groups into gelatin structure substantially enhanced the mucoadhesive performance of all formulations based on Gel-IA, Gel-CA, and Gel-MA microparticles. The ability of chemically modified gelatin samples to adhere well to the mucosal tissue is related to the reaction of unsaturated functional groups with thiol groups in cysteine-rich subdomains present in mucin via thiol–ene click Michael-type addiction reaction to form covalent bonds, which occurs under physiologically relevant conditions (Figure 7). This is a quick process that allows establishing improved adhesion within a reasonable period following dosage form administration on the mucosal surface. Additionally, the residual protonated primary amine groups within the modified gelatin structure could also bind with mucins through electrostatic interactions as pH of ANF solution used is weakly acidic (pH 5.80).

The microparticles composed of cross-linked gelatin and derivatives exhibited poorer mucoadhesive properties compared to the particles prepared with non-cross-linked polymers. This indicates that diffusivity of macromolecules plays a substantial role in mucoadhesion. Cross-linked macromolecules cannot diffuse freely and form an interpenetrating layer with the mucus, which reduces the ability of microparticles to adhere to mucosal surface. This finding is in good agreement with the diffusion theory of mucoadhesion.^10^

## 4 Conclusions

In this study, we report the synthesis and characterization of crotonoylated, itaconoylated, and methacryloylated gelatin (Gel-CA, Gel-IA, and Gel-MA, respectively) derivatives with enhanced mucoadhesive properties. The modification was confirmed using ^1^H NMR, FTIR spectroscopic techniques and TNBSA assay and the degree of functionalization was calculated. The effect of modification on isoelectric point, viscosity and thermo-reversible gelation characteristics of gelatins were also studied. All derivatized gelatins exhibited superior mucoadhesive properties compared to native gelatin. The incorporation of unsaturated anhydrides into gelatins is not detrimental for their toxicological characteristics as evaluated using *in vivo* SMIT assay and *in vitro* MTT assay in HPF cells line. Modified gelatins with unsaturated functional groups could be considered as novel excipients with enhanced mucoadhesive properties for potential formulation of mucoadhesive dosage forms for vaginal and nasal drug delivery. Gel-CA, Gel-IA, and Gel-MA could also find applications in other areas of transmucosal drug delivery, for instance, when formulated as films, gels, micro-, or nanoparticles.

## Supporting Information Available

Feed ratios for the synthesis of crotonoylated, itaconoylated, or methacryloylated gelatin derivatives; Composition of vaginal fluid simulant (VFS) and artificial nasal fluid (ANF); A glycine standard curve used to determine the amount of free amino groups in modified and unmodified gelatins; A fluorescein standard curve; ^1^H NMR spectra of unsaturated anhydrides; ^1^H NMR and FTIR spectra of native gelatin and its modified derivatives; Scanning electron microscopy (SEM) images of spray-dried microparticles based on modified and unmodified gelatin samples; Specific viscosity versus pH curves to determine isoelectric points; Storage and loss moduli curves used to determine thermo-reversible gelation points; Schematic illustration of slug mucosal irritation test (SMIT) procedure using *Arion lusitanicus* species; Exemplar photographs of mucus production by *Arion lusitanicus* slugs in contact with test materials; Exemplar detachment profiles of test materials (PDF)

## Abbreviations

ANF: artificial nasal fluid
BAC: benzalkonium chloride
CA: crotonic anhydride
D_2_O: deuterium oxide
DoF: degree of functionalization
DMEM: Dulbecco’s modified eagle medium
DMSO: dimethyl sulfoxide
FBS: fetal bovine serum
NaFI: fluorescein sodium salt
Gel: gelatin
Gel-CA: crotonoylated gelatin
Gel-IA: itaconoylated gelatin
Gel-MA: methacryloylated gelatin
IA: itaconic anhydride
MA: methacrylic anhydride
MP: mucus production
MTT reagent: 3-(4,5-dimethylthiazol-2-yl)-2,5-diphenyltetrazolium bromide
PBS: phosphate-buffered saline
SMIT: slug mucosal irritation test
TNBSA assay: 2,4,6-trinitrobenzenesulfonic acid assay
VFS: vaginal fluid simulant
