# Supplementary material for: Enhancing Mucoadhesive Properties of Gelatin through Chemical Modification with Unsaturated Anhydrides
Source: Biomacromolecules. 2024 Feb 6;25(3):1612–28. doi: 10.1021/acs.biomac.3c01183 (PMC10934270; doi:10.1021/acs.biomac.3c01183)
Supplement: Supplementary file 1 — bm3c01183_si_001.pdf [file bm3c01183_si_001.pdf]

SUPPORTING INFORMATION

Enhancing Mucoadhesive Properties of Gelatin through Chemical Modification with Unsaturated Anhydrides

Elvira O. Shatabayeva,[1,2] Daulet B. Kaldybekov,[1,2,3,] Leila Ulmanova,[4] Balnur A. Zhaisanbayeva,[5] Ellina A. Mun,[4] Zarina A. Kenessova,[2] Sarkyt E. Kudaibergenov,[3] Vitaliy V. Khutoryanskiy[1,]**

[1] Reading School of Pharmacy, University of Reading, Whiteknights, RG6 6DX Reading, United Kingdom

[2] Department of Chemistry and Chemical Technology, Al-Farabi Kazakh National University, 050040 Almaty, Kazakhstan

[3] Institute of Polymer Materials and Technology, 050019 Almaty, Kazakhstan

[4] School of Sciences and Humanities, Nazarbayev University, 010000 Astana, Kazakhstan

[5] School of Engineering and Digital Sciences, Nazarbayev University, 010000 Astana, Kazakhstan

***Corresponding authors:**

Prof. Vitaliy Khutoryanskiy
E-mail: v.khutoryanskiy@reading.ac.uk
Phone: +44(0) 118 378 6119
Fax: +44(0) 118 378 4703

Dr. Daulet Kaldybekov
E-mail: dauletchem@gmail.com
d.kaldybekov@reading.ac.uk

Materials

Acetic acid, calcium chloride dihydrate ($CaCl_2 \times 2H_2O$), calcium hydroxide ($Ca(OH)_2$), potassium chloride (KCl), and sodium chloride (NaCl) were purchased from Fisher Scientific (Loughborough, UK). Bovine serum albumin (BSA), glucose, glycerol, 1 M hydrochloric acid solution (HCl), lactic acid, potassium hydroxide (KOH), and urea were purchased from Sigma-Aldrich (Gillingham, UK).

Table S1. Feed ratios for the synthesis of crotonoylated, itaconoylated, or methacryloylated gelatin derivatives.

Parameters	Gel-CA	Gel-IA	Gel-MA
Amount of gelatin	0.5 g	0.5 g	0.5 g
Amount of anhydride*	0.05 mL (0.337 mmol)	0.05 mL (0.562 mmol)	0.05 mL (0.335 mmol)
	0.1 mL (0.675 mmol)	0.1 mL (1.124 mmol)	0.1 mL (0.671 mmol)
	0.2 mL (1.350 mmol)	0.2 mL (2.248 mmol)	0.2 mL (1.343 mmol)

*The amount of anhydride was varied with respect to the free amino groups (0.434 mmol) per gram of gelatin (from porcine skin, type A). Gel, gelatin; CA, crotonic anhydride; IA, itaconic anhydride; MA, methacrylic anhydride.

Table S2. Composition of vaginal fluid simulant (VFS).

Compounds	Amounts (g)
NaCl	3.51 g (60 mmol)
KOH	1.40 g (25 mmol)
$Ca(OH)_2$	0.222 g (3 mmol)
BSA	0.018 g
Lactic acid	2.0 g (22 mmol)
Acetic acid	1.0 g (11 mmol)
Glycerol	0.16 g (2 mmol)
Urea	0.40 g (7 mmol)
Glucose	5.0 g (28 mmol)

A vaginal fluid simulant (VFS) was prepared as reported in a previous protocol.[1] The above ingredients were dissolved in deionized water. The solution was left stirring overnight at room temperature and then the pH was adjusted to pH 4.0 with 1 M HCl before making the total volume to 1 L. VFS solution was kept at 37 °C throughout the experiments using a water bath.

Table S3. Composition of artificial nasal fluid (ANF).

Compounds	Amounts (g)
NaCl	7.45 g (127 mmol)
KCl	1.29 g (17 mmol)
$CaCl_2 \times 2H_2O$	0.32 g (2.2 mmol)

Artificial nasal fluid (ANF) was prepared according to an established protocol.[2–4] The above ingredients were dissolved in deionized water. The solution was left stirring at room temperature until the compounds fully dissolved, then the pH was adjusted to pH 5.80 with 1 M HCl before making the total volume to 1 L. ANF solution was kept at 37 °C throughout the experiments using a water bath.

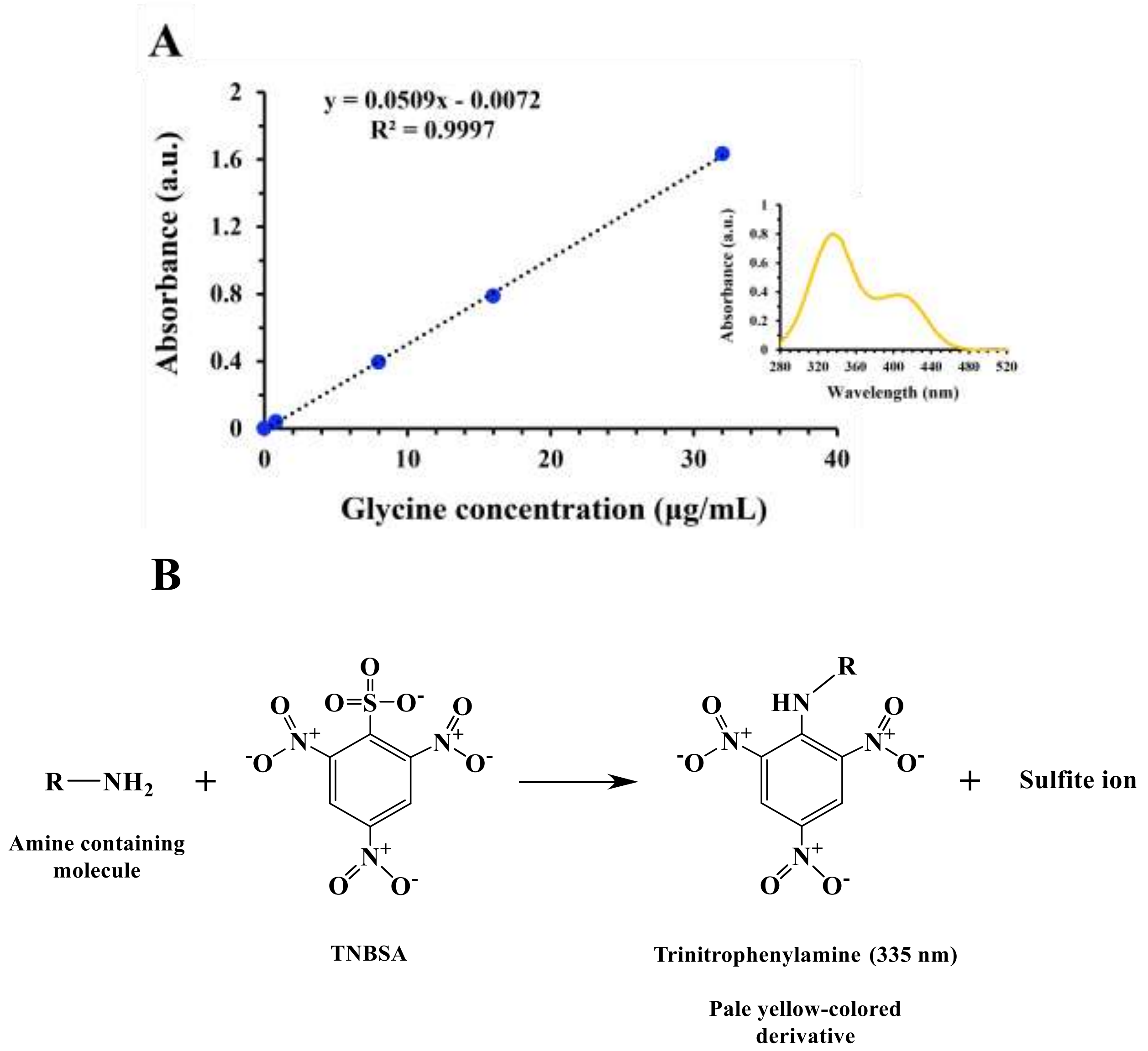

Figure S1. (A): A glycine standard curve used to determine the amount of free amino groups in modified and unmodified gelatins. Insert: UV-Vis spectrum of 2,4,6-trinitrobenzene sulfonic acid (TNBSA) + glycine reaction product. (B): TNBSA is a rapid and sensitive assay reagent for the determination of free amino groups. Primary amines, upon reaction with TNBSA, form a highly chromogenic derivative, which can be measured at 335 nm.[5]

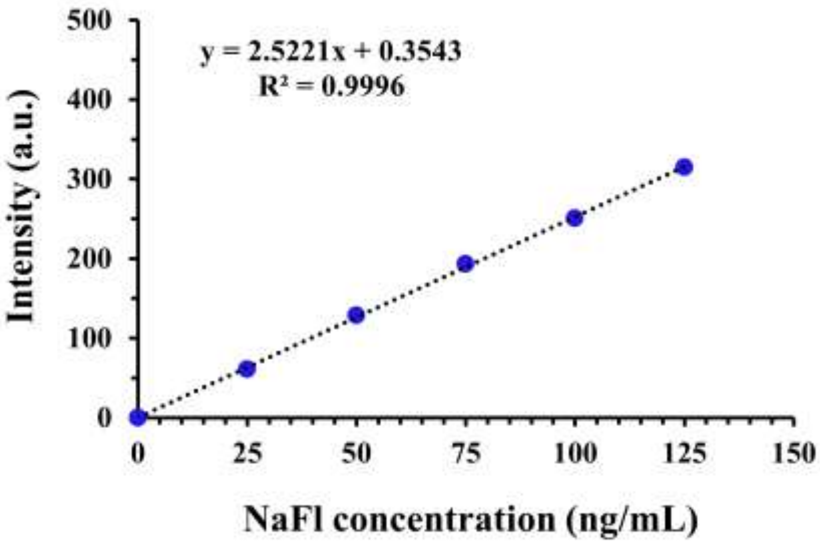

Figure S2. Fluorescein standard curve used to determine the amount of non-crosslinked NaFl-loaded modified and unmodified gelatin microparticles washed off the sheep nasal mucosa during *ex vivo* mucoadhesion experiments.

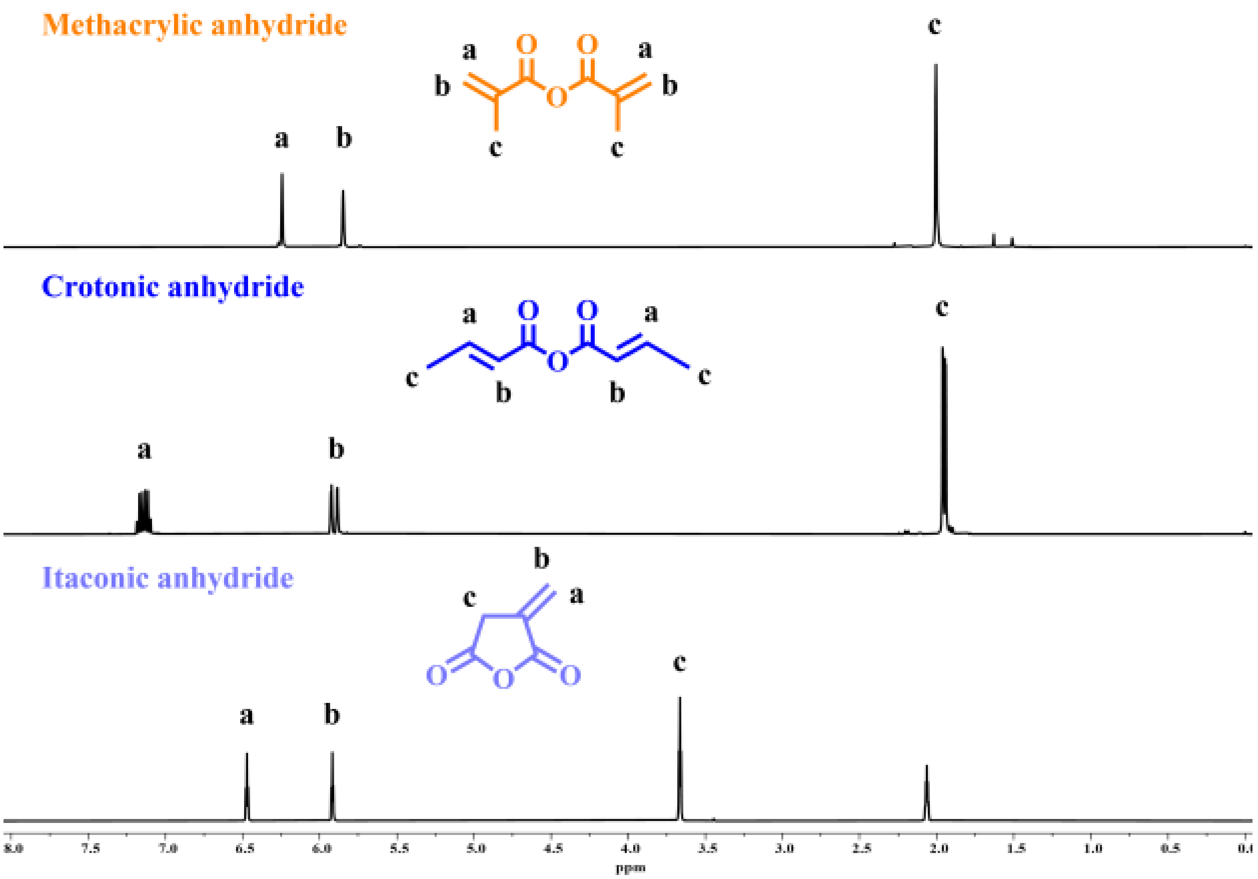

Figure S3. ^{1}H NMR spectra of methacrylic anhydride (top), crotonic anhydride (center), and itaconic anhydride (bottom) recorded in deuterated chloroform ($CDCl_3$).

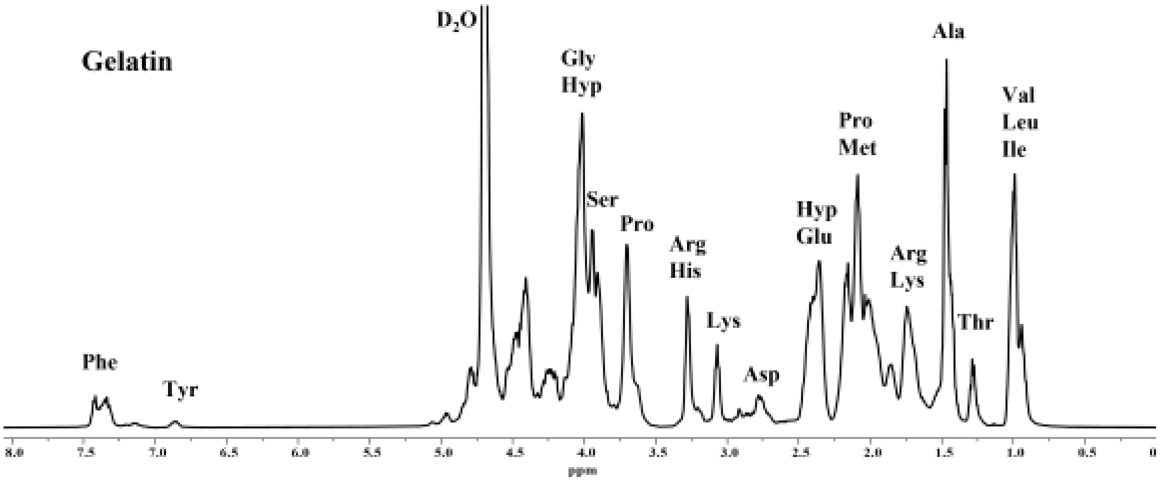

Figure S4. ^{1}H NMR spectrum of gelatin from porcine skin (Type A, 175 g Bloom) recorded in deuterium oxide (D_2O) at 37 °C. Location of the peaks according to the literature reported.[6,7]

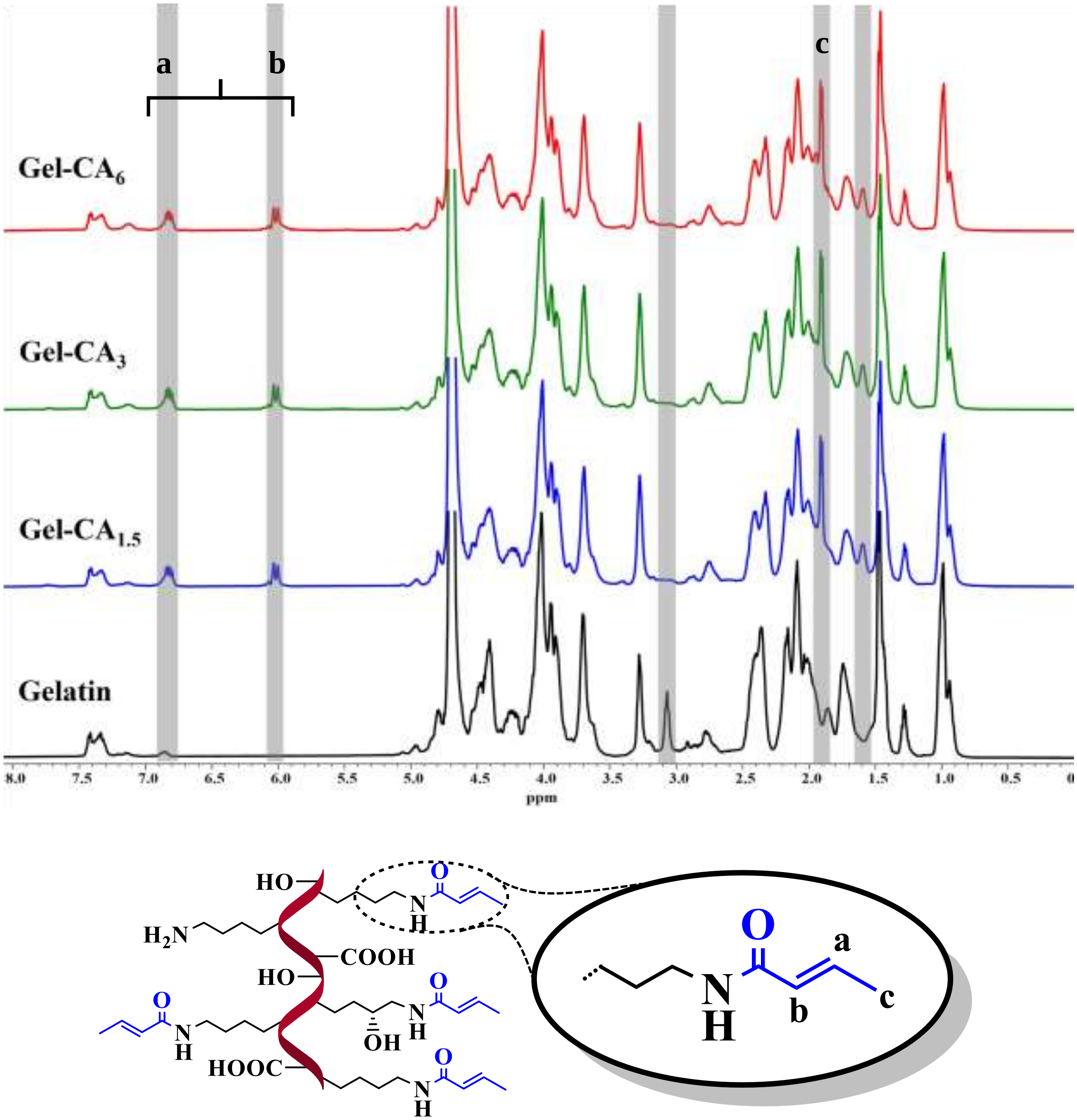

Figure S5. ^{1}H NMR spectra of native gelatin and crotonoylated gelatins (Gel-CA) recorded in deuterium oxide (D_2O) at 37 °C.

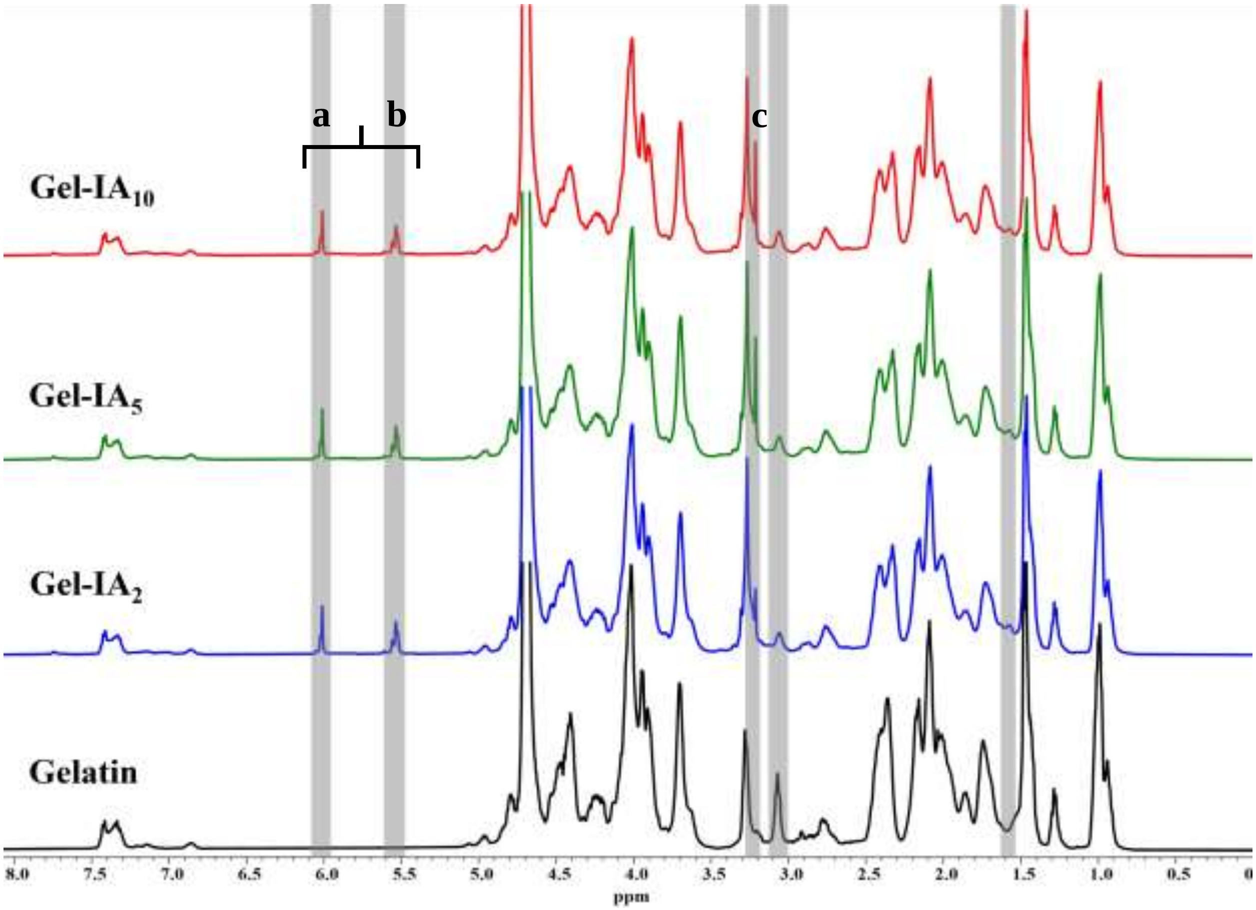

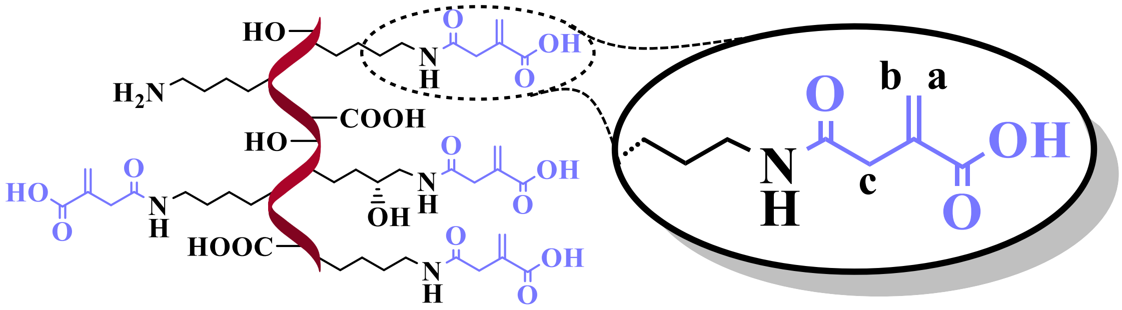

Figure S6. ^{1}H NMR spectra of native gelatin and itaconoylated gelatins (Gel-IA) recorded in deuterium oxide (D_2O) at 37 °C.

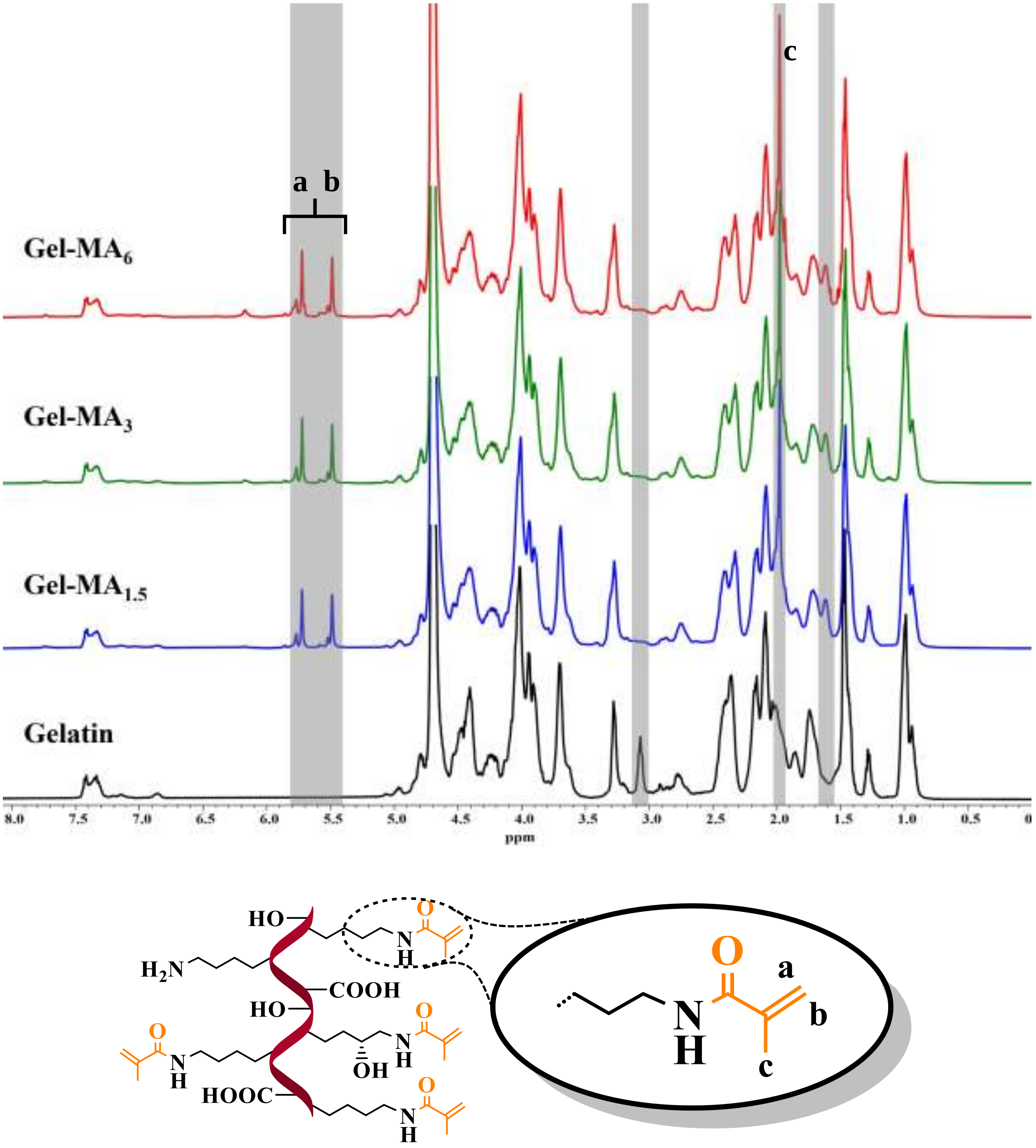

Figure S7. ^{1}H NMR spectra of native gelatin and methacryloylated gelatins (Gel-MA) recorded in deuterium oxide (D_2O) at 37 °C.

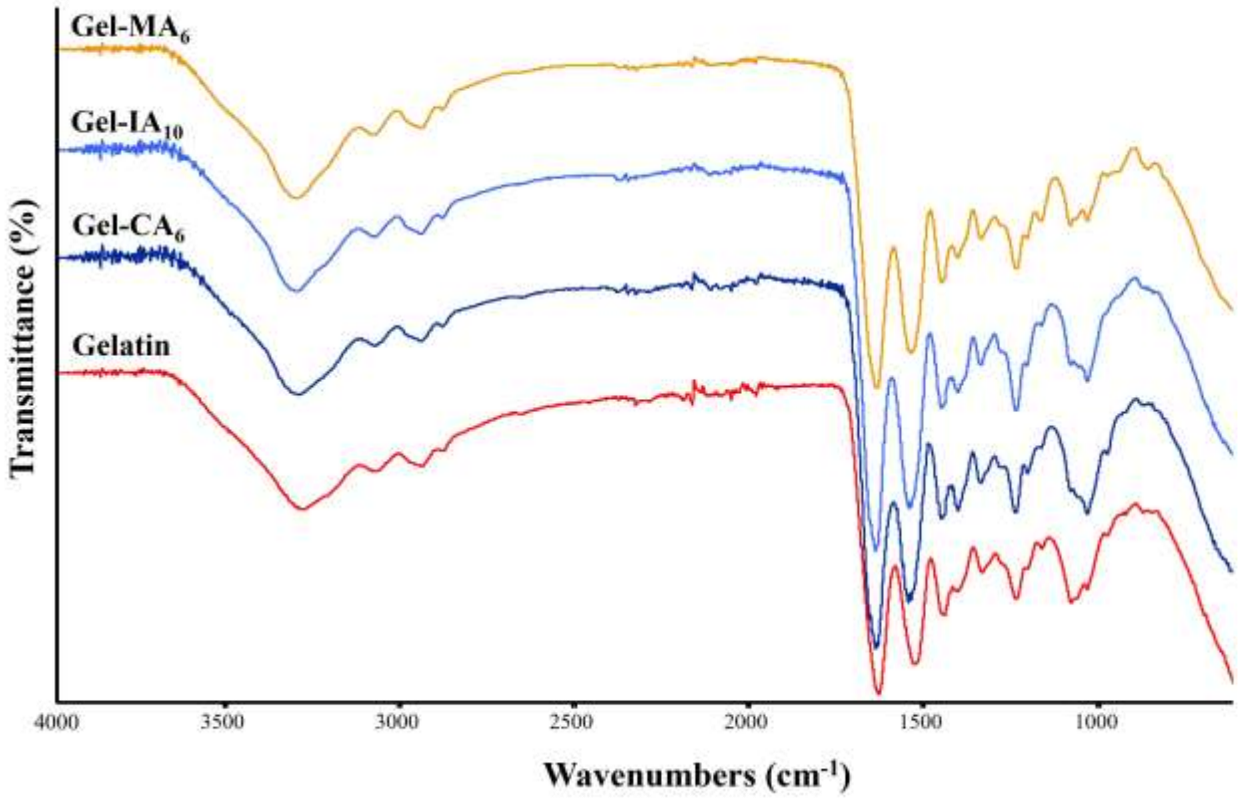

Figure S8. FTIR spectra of native gelatin and its crotonoylated (Gel-CA_6), itaconoylated (Gel-IA_{10}), and methacryloylated (Gel-MA_6) derivatives.

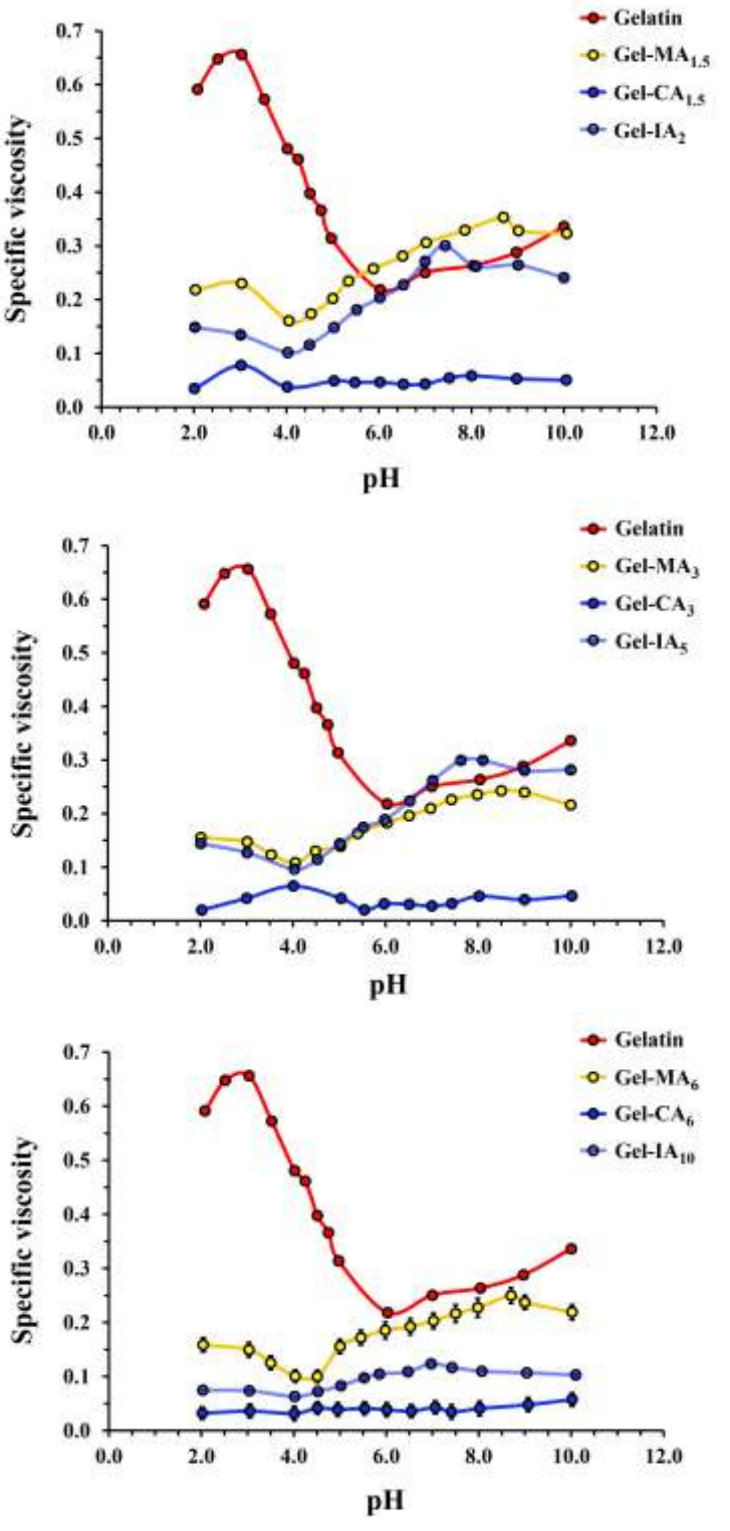

Figure S9. Determination of the IEPviscometry of native gelatin and its chemically modified derivatives (at different molar excess of anhydrides) using a viscometric technique. Gel-MA, methacryloylated gelatin; Gel-CA, crotonoylated gelatin; Gel-IA, itaconoylated gelatin.

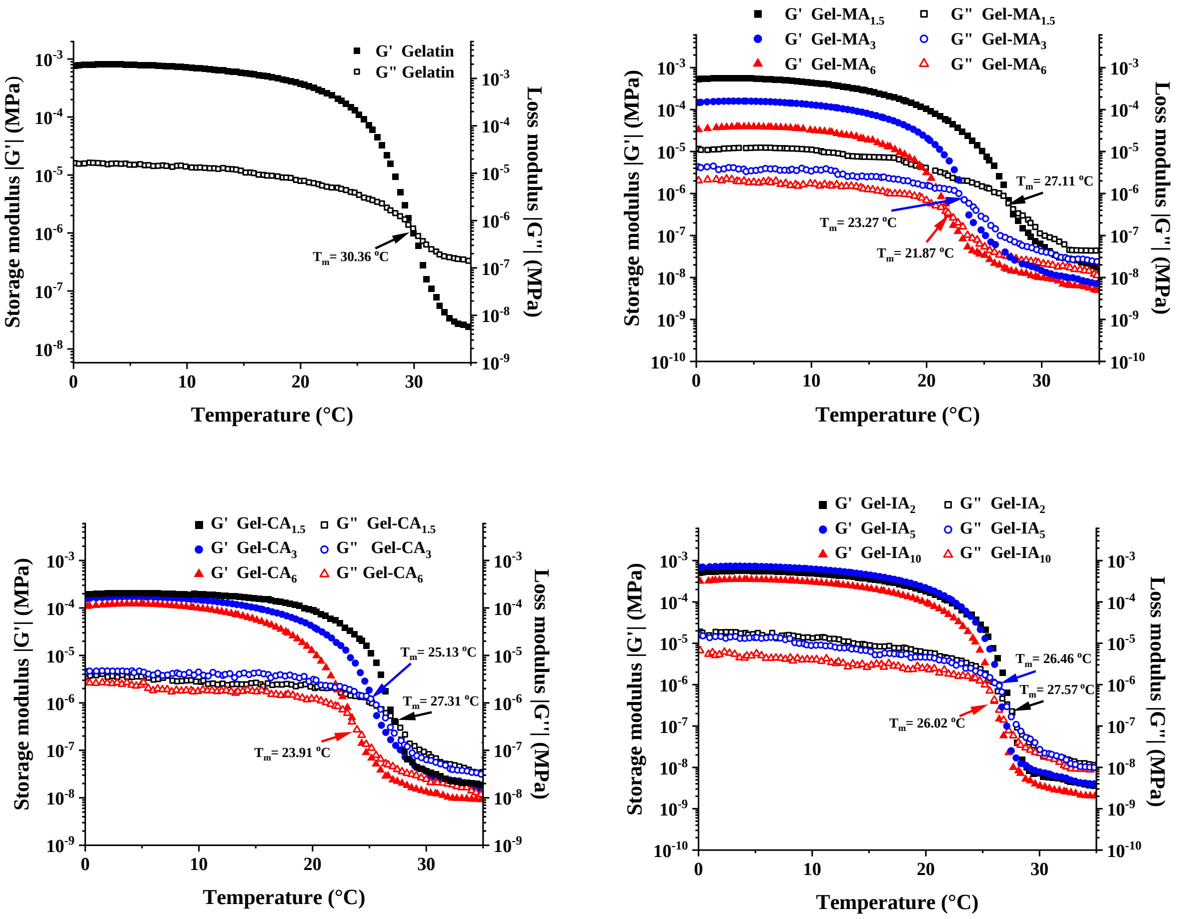

Figure S10. Rheological heating (0–40 °C) scans of modified and unmodified gelatin samples. The melting temperature (T_m) was determined from where the storage (G′) and loss (G″) moduli intersect upon the heating period. Gel-MA, methacryloylated gelatin; Gel-CA, crotonoylated gelatin; Gel-IA, itaconoylated gelatin. Note, for clarity and convenience, the temperature range presented was cut off in some figures.

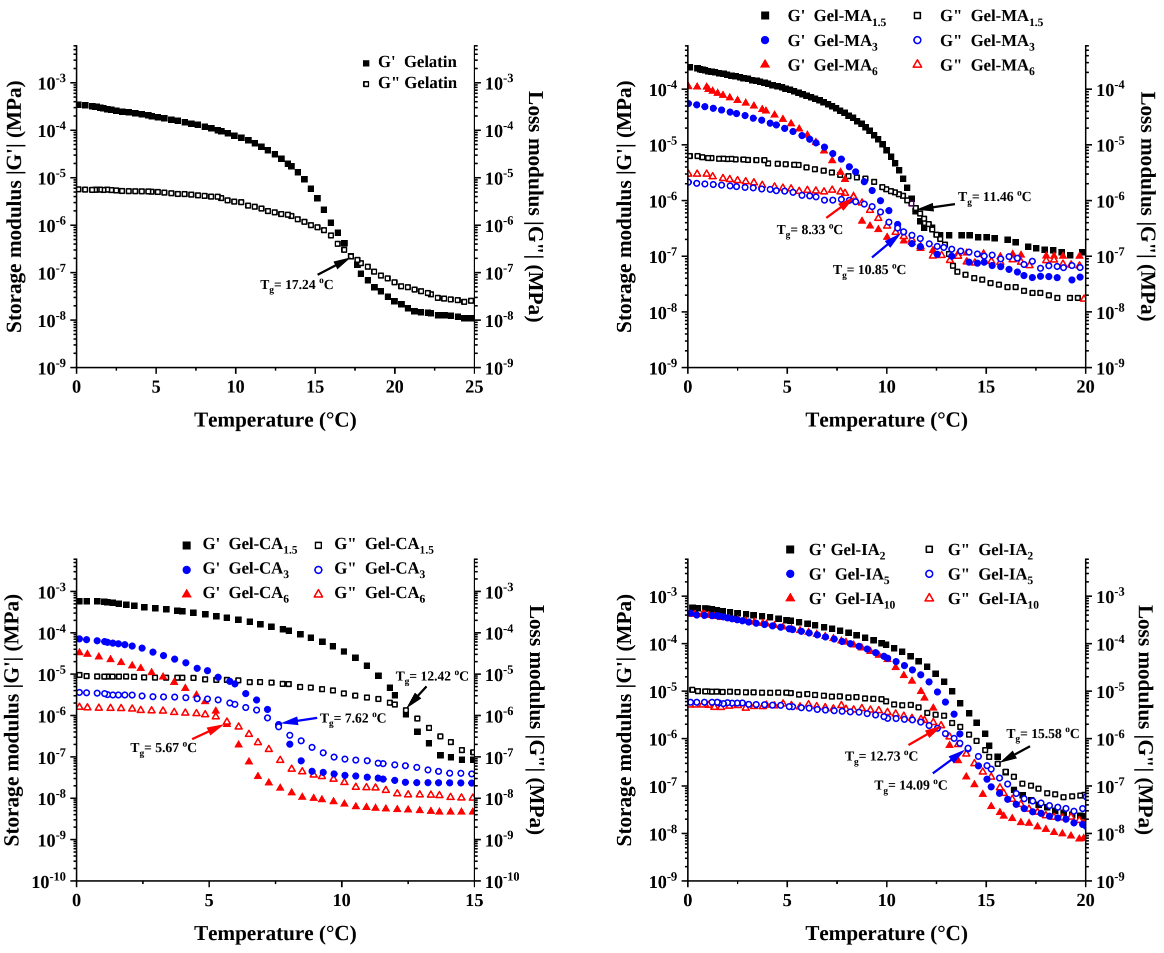

Figure S11. Rheological cooling (40–0 °C) scans of modified and unmodified gelatin samples. The gelation temperature (T_{gel}) was determined from where the storage (G′) and loss (G″) moduli intersect upon the cooling period. Gel-MA, methacryloylated gelatin; Gel-CA, crotonoylated gelatin; Gel-IA, itaconoylated gelatin. Note, for clarity and convenience, the temperature range presented was cut off in some figures.

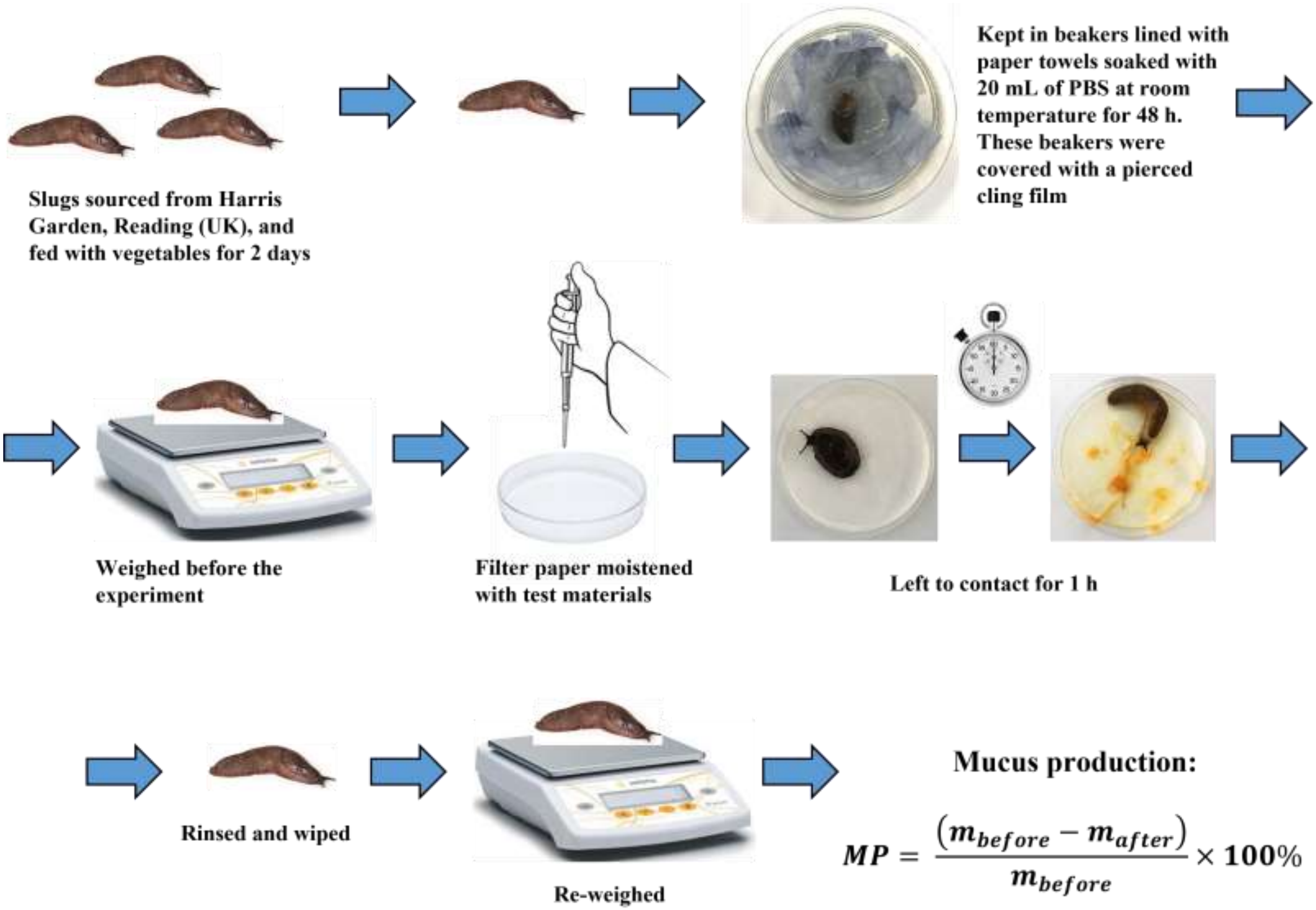

Figure S12. Schematic illustration of step-by-step slug mucosal irritation test (SMIT) procedure using *Arion lusitanicus* species. MP: mucus production expressed as a % of the initial body weight loss of the slugs.

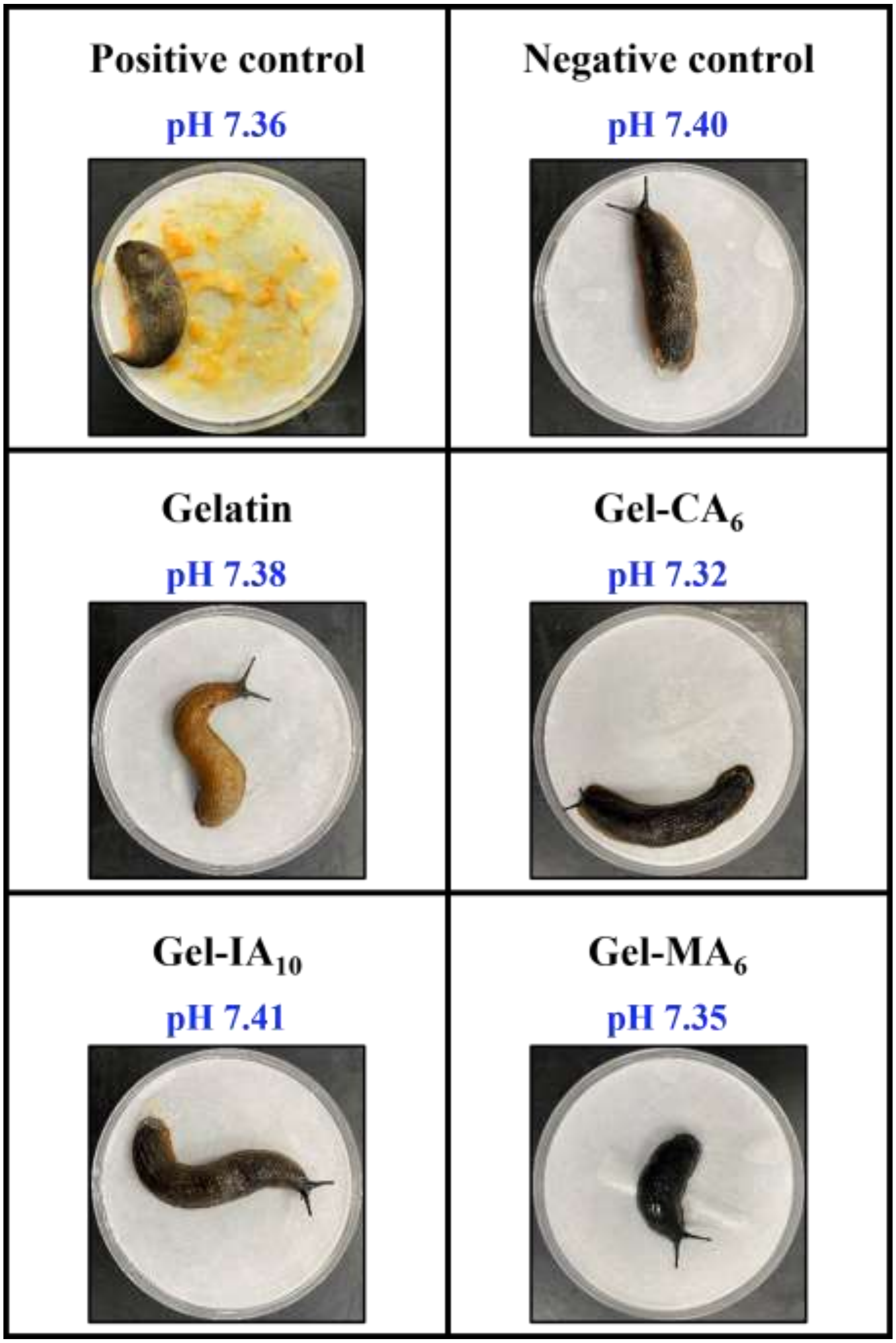

Figure S13. Exemplar photographs of mucus production (MP) by *Arion lusitanicus* slugs in contact with positive (1% w/v BAC in PBS) and negative (PBS solution) controls as well as with gelatin-based test materials (each prepared at 1.3% w/v in PBS) after 60 min exposure. BAC, benzalkonium chloride; Gel-CA_6, crotonoylated gelatin; Gel-IA_{10}, itaconoylated gelatin; Gel-MA_6, methacryloylated gelatin; PBS, phosphate-buffered saline.

Non-crosslinked

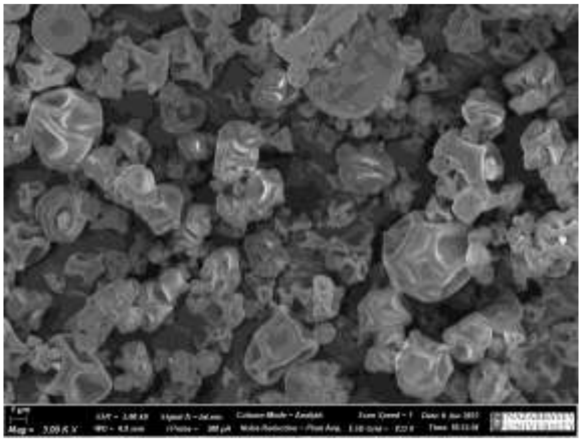

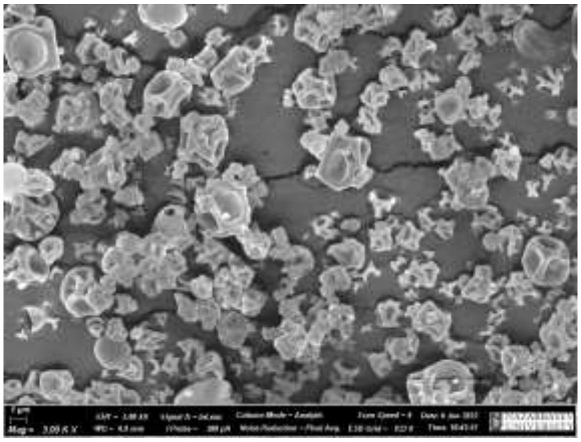

Gelatin | **Crotonoylated gelatin**

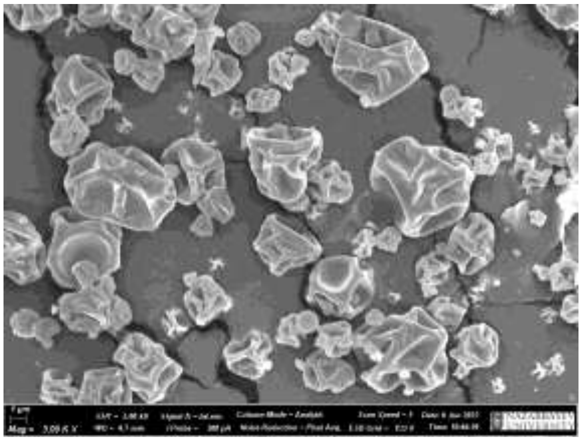

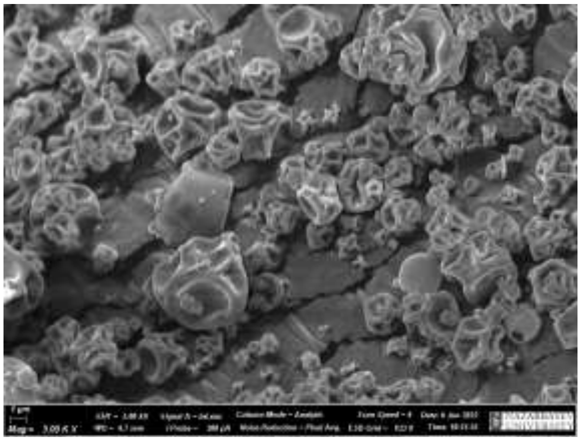

Itaconoylated gelatin | **Methacryloylated gelatin**

Figure S14. Scanning electron microscopy (SEM) images were acquired for spray-dried microparticles of both modified and unmodified non-crosslinked gelatins. The SEM micrographs were observed under × 3,000 magnification, scale bars = 1 μm.

Crosslinked

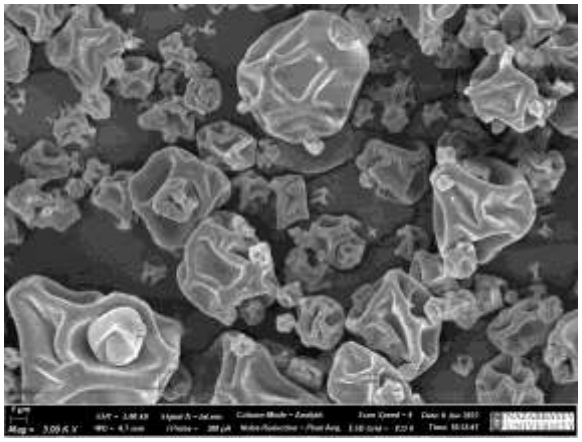

Gelatin

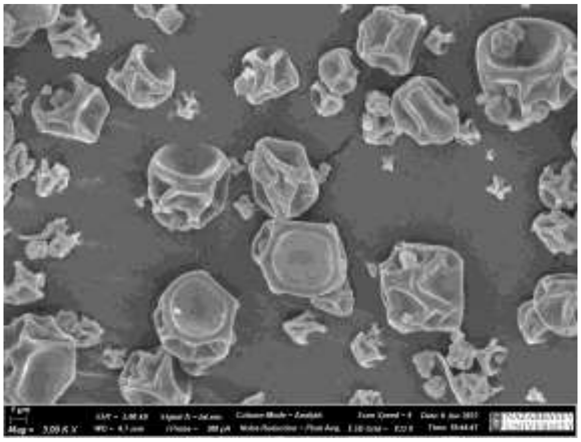

Crotonoylated gelatin

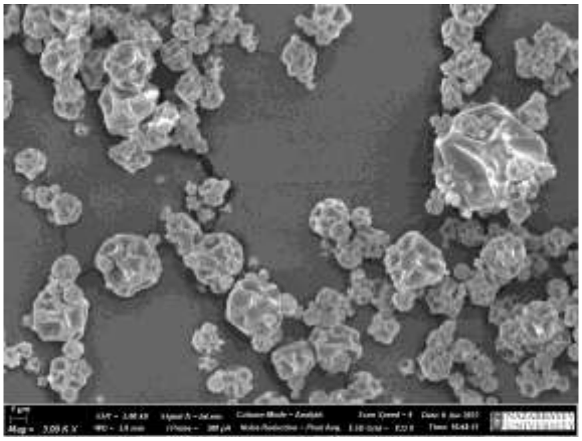

Itaconoylated gelatin

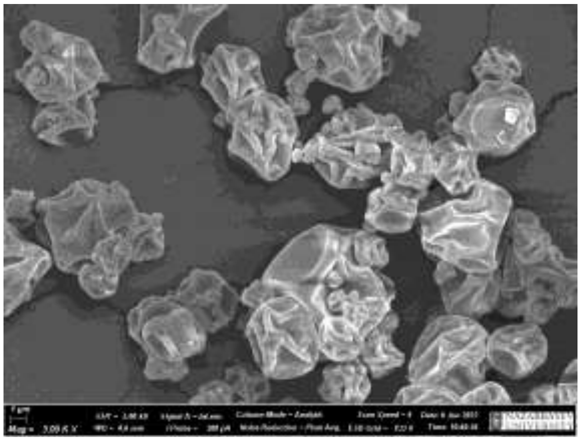

Methacryloylated gelatin

Figure S15. Scanning electron microscopy (SEM) images were acquired for spray-dried microparticles of both modified and unmodified crosslinked gelatins. The SEM micrographs were observed under × 3,000 magnification, scale bars = 1 μm.

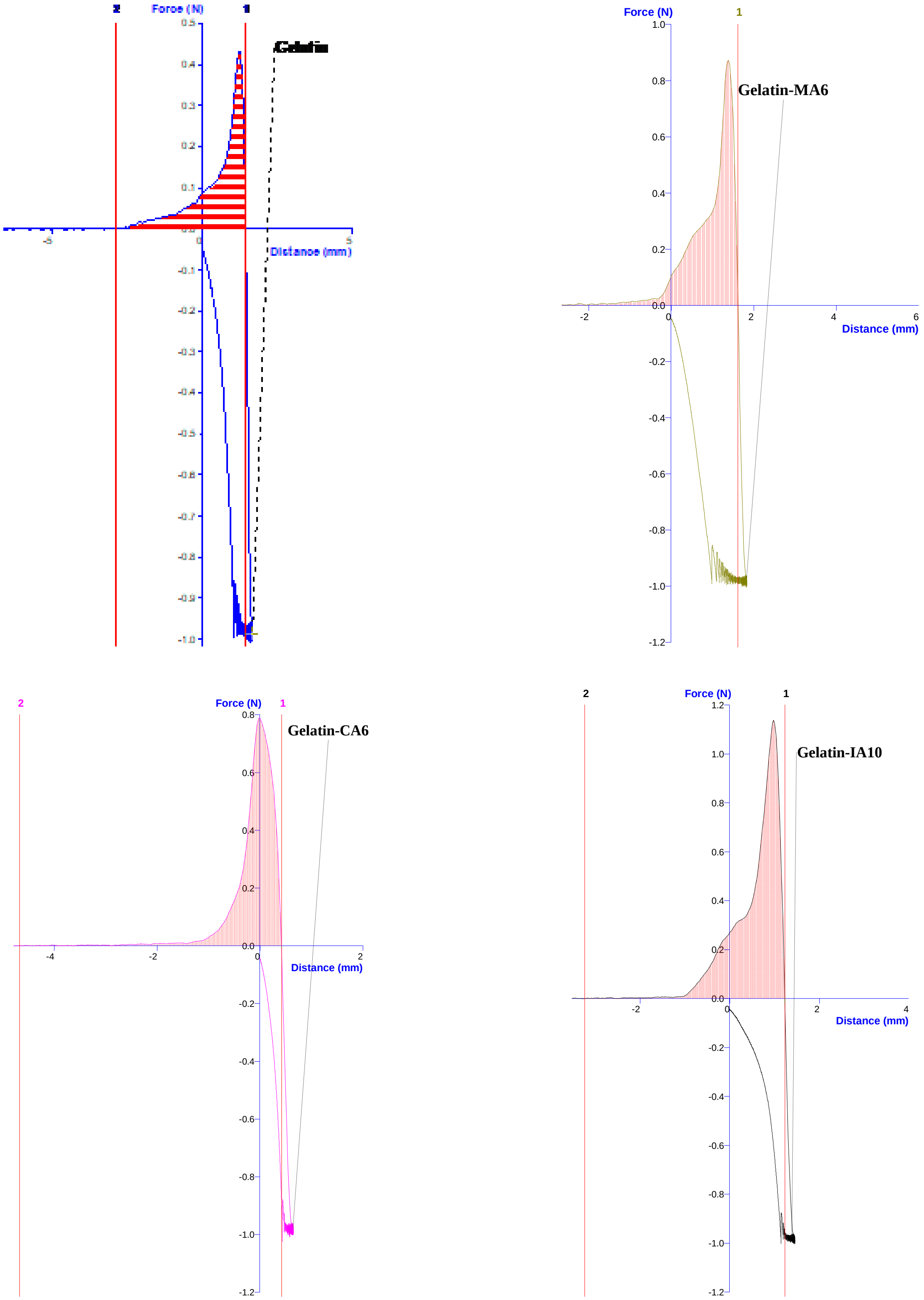

Figure S16. Exemplar detachment profiles of non-crosslinked microparticles based on gelatin and Gel-MA_6, methacryloylated gelatin; Gel-CA_6, crotonoylated gelatin and Gel-IA_{10}, itaconoylated gelatin from freshly excised sheep nasal mucosa.

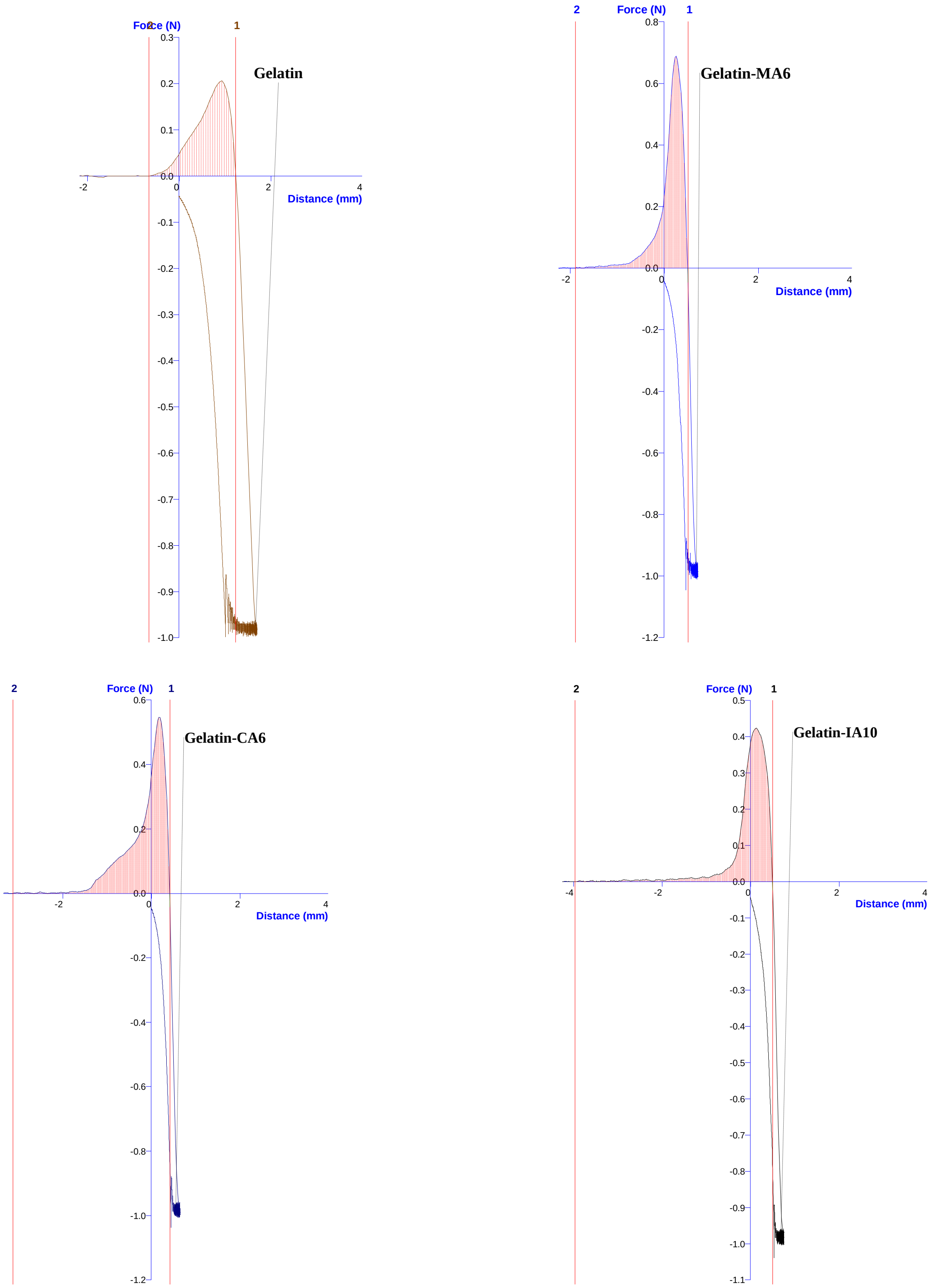

Figure S17. Exemplar detachment profiles of crosslinked microparticles based on gelatin and Gel-MA_6, methacryloylated gelatin; Gel-CA_6, crotonoylated gelatin and Gel-IA_{10}, itaconoylated gelatin from freshly excised sheep nasal mucosa.

References

(1) Owen, D. H.; Katz, D. F. A Vaginal Fluid Simulant. *Contraception* **1999**, *59* (2), 91–95.

(2) da Silva Barbi, M.; Carvalho, F. C.; Kiill, C. P.; da Silva Barud, H.; Santagneli, S. H.; Ribeiro, S. J. L.; Gremião, M. P. D. Preparation and Characterization of Chitosan Nanoparticles for Zidovudine Nasal Delivery. *J. Nanosci. Nanotechnol.* **2015**, *15* (1), 865–874.

(3) Porfiryeva, N. N.; Nasibullin, S. F.; Abdullina, S. G.; Tukhbatullina, I. K.; Moustafine, R. I.; Khutoryanskiy, V. V. Acrylated Eudragit® E PO as a Novel Polymeric Excipient with Enhanced Mucoadhesive Properties for Application in Nasal Drug Delivery. *Int. J. Pharm.* **2019**, *562*, 241–248.

(4) Porfiryeva, N. N.; Semina, I. I.; Salakhov, I. A.; Moustafine, R. I.; Khutoryanskiy, V. V. Mucoadhesive and Mucus-Penetrating Interpolyelectrolyte Complexes for Nose-to-Brain Drug Delivery. *Nanomedicine Nanotechnology, Biol. Med.* **2021**, *37*, 102432.

(5) Habeeb, A. F. S. A. Determination of Free Amino Groups in Proteins by Trinitrobenzenesulfonic Acid. *Anal. Biochem.* **1966**, *14* (3), 328–336.

(6) Billiet, T.; Gasse, B. Van; Gevaert, E.; Cornelissen, M.; Martins, J. C.; Dubruel, P. Quantitative Contrasts in the Photopolymerization of Acrylamide and Methacrylamide-Functionalized Gelatin Hydrogel Building Blocks. *Macromol. Biosci.* **2013**, *13* (11), 1531–1545.

(7) Norris, S. C. P.; Delgado, S. M.; Kasko, A. M. Mechanically Robust Photodegradable Gelatin Hydrogels for 3D Cell Culture and in Situ Mechanical Modification. *Polym. Chem.* **2019**, *10* (23), 3180–3193.
